# Novel ^13^C enrichment technique reveals early turnover of DHA in peripheral tissues

**DOI:** 10.1016/j.jlr.2023.100357

**Published:** 2023-03-21

**Authors:** Brinley J. Klievik, Adam H. Metherel, Giulia Cisbani, Rodrigo Valenzuela, Richard P. Bazinet

**Affiliations:** 1 Department of Nutritional Sciences, Temerty Faculty of Medicine, University of Toronto, 1 King’s College Circle, Toronto, Ontario, Canada; 2 Department of Nutrition, Faculty of Medicine, University of Chile, Santiago, Chile

**Keywords:** fatty acid metabolism, omega-3 fatty acids, DHA, diet, brain, liver, mass spectrometry

## Abstract

The brain is rich in DHA, which plays important roles in regulating neuronal function. Recently, using compound-specific isotope analysis that takes advantage of natural differences in carbon-13 content (^13^C/^12^C ratio or δ^13^C) of the food supply, we determined the brain DHA half-life. However, because of methodological limitations, we were unable to capture DHA turnover rates in peripheral tissues. In the current study, we applied compound-specific isotope analysis *via* high-precision GC combustion isotope ratio mass spectrometry to determine half-lives of brain, liver, and plasma DHA in mice following a dietary switch experiment. To model DHA tissue turnover rates in peripheral tissues, we added earlier time points within the diet switch study and took advantage of natural variations in the δ^13^C-DHA of algal and fish DHA sources to maintain DHA pool sizes and used an enriched (uniformly labeled ^13^C) DHA treatment. Mice were fed a fish-DHA diet (control) for 3 months, then switched to an algal-DHA treatment diet, the ^13^C enriched-DHA treatment diet, or they stayed on the control diet for the remainder of the study time course. In mice fed the algal and ^13^C enriched-DHA diets, the brain DHA half-life was 47 and 46 days, the liver half-life was 5.6 and 7.2 days, and the plasma half-life was 4.7 and 6.4 days, respectively. By using improved methodologies, we calculated DHA turnover rates in the liver and plasma, and our study for the first time, by using an enriched DHA source (very high δ^13^C), validated its utility in diet switch studies.

DHA (22:6n-3) is one type of omega-3 (n-3) PUFA, the other two common types being α-linolenic acid (ALA, 18:3n-3) and EPA (20:5n-3). ALA is found in many plant seed oils, such as canola, soybean, and flaxseed, and DHA and EPA are found mainly in seafood and fish sources (1). The brain is rich in PUFAs, particularly DHA, which represents approximately 40% of total brain PUFAs (2). DHA plays critical roles in neuronal survival, neurogenesis, synaptic function, and the regulation of brain inflammation (3, 4). It is therefore suggested that a deficiency in DHA is linked to many neurological and psychiatric disorders such as Parkinson’s, Alzheimer’s, schizophrenia, and major depressive disorder. Although DHA appears to be crucial in supporting brain function, the conversion of its dietary precursor ALA to DHA in the brain is low, making the brain reliant upon the uptake of preformed DHA from plasma and tissue pools to replace what has been metabolized (3, 4, 5, 6).

There are several different tissue pools supplying the brain with DHA such as the liver, which is the site of DHA synthesis starting from its precursor, ALA, via multiple desaturation, elongation, and β-oxidation steps (7). DHA is then esterified to phospholipids, triacylglycerols (TAGs), or cholesteryl esters and secreted as lipoproteins into the bloodstream. DHA can also be sequestered into peripheral tissue, like adipose, where it can be released into the circulation as NEFA available for uptake to the brain (8). From adipose tissue, lipolysis of DHA could potentially provide a long-term supply of DHA for the brain (8).

Isotope tracers have been useful when studying brain fatty acid kinetics, but prohibitive costs often limit these studies to acute study durations and single-dose administrations (7). Our laboratory has developed an alternative and cost-effective technique, called compound-specific isotope analysis (CSIA) that takes advantage of natural differences in carbon-13 content (^13^C/^12^C ratio or δ^13^C) of the food supply to better understand tissue DHA metabolism. CSIA can pinpoint the origin of a specific compound of interest since the carbon isotopic makeup of a molecule is conserved following incorporation from the diet (4). Importantly, natural variations in the photosynthetic pathways of C3 and C4 plants along with marine fish sources result in different isotopic signatures, which can be used to measure tissue turnover rates (4, 9). The C3 photosynthetic pathway discriminates against carbon-13; therefore, it has low carbon-13 in tissues (−20 to −32 milliUrey [mUr]; where 1 mUr equals 1 part per thousand [‰] or 0.1% ^13^C–^12^C). The C4 photosynthetic pathway does not discriminate against carbon-13; therefore, it has higher carbon-13 in tissues (−9 to −17 mUr) (10). Moreover, marine-based aquatic organisms yield more intermediate carbon-13 in fatty acids (−18 to −30 mUr) (10). Furthermore, because of lakes and rivers having less dissolved carbon pools, freshwater-based aquatic phytoplankton appear have less enriched δ^13^C values than their marine counterparts ranging from −42 to −26 mUr (10).

Previously, we applied CSIA to determine the half-life of brain DHA in mice following a diet switch experiment using different n-3 PUFA sources (ALA and DHA). These brain half-lives determined by CSIA fit estimates from kinetic isotope tracer studies, which ensured the feasibility of CSIA to study the metabolism of fatty acids without the use of isotopically labeled fatty acid tracers. However, this model was limited by *1*) the selection of time points that missed the window of DHA turnover in peripheral tissues and *2*) the lack of steady-state DHA pool sizes in peripheral tissues limiting accurate DHA turnover estimates. In the current study, we included more frequent and earlier time points to better model DHA tissue turnover in peripheral tissues, assessed DHA turnover more accurately by maintaining DHA pool sizes via DHA switching from fish (low δ^13^C) to algal (high δ^13^C) sources, and in addition, used an enriched DHA source (very high δ^13^C) to validate its utility in diet switch studies.

## MATERIALS AND METHODS

### Materials

The fatty acid internal standard, docosatrienoic acid (22:3n-3) ethyl ester, GC reference standard (GLC-462), fish oil ethyl DHA, and microalgal ethyl DHA were purchased from NuChek Prep, Inc (Elysian, MN). Uniformly labeled ^13^C DHA methyl ester was purchased from Cambridge Isotope Laboratories, Inc. For isotopic analysis, the reference materials (USGS70, USGS71, and USGS72) were obtained from Reston Stable Isotope Laboratory-United States (Reston, VA). Boron trifluoride in methanol (14%) was purchased from Sigma-Aldrich. All solvents used were American Chemical Society or HPLC grade and were purchased from either MilliporeSigma (Mississauga, ON, Canada) or Fisher Scientific (Ottawa, ON, Canada).

### Animals

The University of Toronto Animal Ethics Committee approved the experimental animal protocol (protocol #20012549), which was conducted in accordance with the policy and guidelines of the Canadian Council on Animal Care and the Regulations of Animals Research Act of Ontario. The animal facility maintained standard conditions in a temperature-controlled (21°C) environment under a 12 h light/12 h dark cycle. Throughout the study, food and water were available ad libitum, and substantial care was taken to minimize animal suffering.

**Fig. 1:**
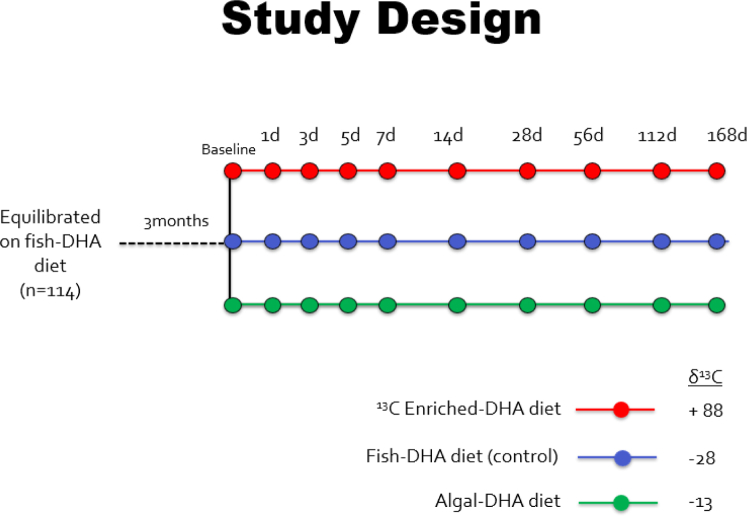
Diet switch experimental study design schematic. About 114 twenty-one-day-old male BALB/c pups were equilibrated to the fish-DHA diet (control) for 3 months to establish a stable brain DHA isotopic signature, prior to the diet switch. From the fish-DHA equilibrated diet group, mice were stratified randomly onto either the algal-DHA treatment diet, the ^13^C enriched-DHA treatment diet, or they stayed on the control diet for the remainder of the study time course. Treatment and control mice (*n* = 4) were sacrificed at 1, 3, 5, 7, 14, 28, 56, 112, and 168 days post diet switch. A group of six mice from the equilibrated control diet was sacrificed prior to diet switch for baseline measurements.

One hundred fourteen 21-day-old BALB/c male pups were ordered, and upon arrival, acclimated for 1 week. The male pups were placed on the fish-DHA (control) diet for 3 months to establish a stable brain DHA isotopic signature. Following 3 months of the fish-DHA diet, mice were randomized to one of three diets: *1*) an algal-DHA diet (algal-DHA), *2*) a ^13^C enriched-DHA diet (^13^C-enriched DHA), or *3*) they remained on the control diet (fish-DHA) (Fig. 1). Post diet switch, the mice were euthanized at 1, 3, 5, 7, 14, 28, 56, 112, and 168 days. A group of six mice from the equilibrated control diet were euthanized prior to the diet switch for baseline measurements. Blood was collected from the left ventricle prior to euthanasia by intracardiac perfusion under isofluorane anesthetic. Brain and liver tissues were collected, flash frozen in liquid nitrogen, and stored at −80°C until biochemical processing.

### Diets

Three DHA diets were formulated from the AIN-93G diet (Dyets, Inc, Bethlehem, PA), each containing DHA from different sources (fish-DHA, algal-DHA, and^13^C enriched-DHA). The diets contained, by weight, 10% fat, 60% carbohydrate, 20% protein, 5% fiber, and 5% vitamins/minerals/essential amino acids. Background oils were derived from safflower, fully hydrogenated coconut oils, and added oils (33.8%, 64.2%, and 2.0% by weight, respectively). All three diets are isocaloric with added oils from three different sources. Added oils were 2% fish oil DHA ethyl ester (Nu-Chek Prep, Inc) for the fish-DHA diet, 2% microalgae source DHA ethyl ester (Nu-Chek Prep, Inc) for the algal-DHA diet, and a mix of 99.75% fish oil DHA ethyl ester and 0.25% ^13^C DHA methyl ester (Cambridge Isotopes laboratory, Inc) for the ^13^C enriched-DHA diet. The added oils for the fish-DHA and algal-DHA diets were sent to Dyets Inc, from the supplier (Nu-Chek Prep, Inc), for diet formulation. The added oils for the ^13^C enriched-DHA diet were delivered and mixed at the University of Toronto, then formulated by Dyets Inc. The fatty acid compositions of experimental diets were measured in our laboratory by GC-flame ionization detection (GC-FID) in triplicate, and δ^13^C-DHA signatures of each diet were measured by GC combustion isotope ratio mass spectrometry (GC/C/IRMS) (Table 1). The carbon isotope ratios of the fish, algal, and ^13^C enriched-DHA diets were determined to be −28.2 ± 0.01, −13.2 ± 0.1, and +87.8 ± 0.8 mUr, respectively.

**Table 1:** Composition of fatty acids and δ^13^C-DHA signatures of each diet

Fatty acid	Relative percent of fatty acids		
	Fish-DHA	Algal-DHA	^13^C Enriched-DHA
10:0	1.7 ± 0.4	1.3 ± 0.5	1.8 ± 0.5
12:0	25.5 ± 0.2	28.2 ± 2.6	28.8 ± 2.2
14:0	12.5 ± 0.3	13.8 ± 0.9	13.7 ± 0.4
15:0	0.1 ± 0.002	0.1 ± 0.002	0.1 ± 0.001
16:0	10.1 ± 0.2	10.3 ± 0.3	10.0 ± 0.3
17:0	ND	ND	ND
18:0	10.3 ± 0.1	9.4 ± 0.4	8.9 ± 0.5
20:0	0.3 ± 0.003	0.2 ± 0.03	0.2 ± 0.1
SFAs	60.5 ± 0.1	63.3 ± 0.3	63.5 ± 0.3
16:1n-7	0.1 ± 0.004	0.1 ± 0.002	0.1 ± 0.002
18:1n-7	ND	ND	ND
18:1n-9	5.9 ± 0.04	5.4 ± 0.4	5.4 ± 0.4
20:1n-9	ND	ND	ND
*MUFAs*	6.0 ± 0.01	5.5 ± 0.2	5.5 ± 0.2
18:2n-6	29.4 ± 0.3	27.3 ± 1.9	27.1 ± 1.8
20:2n-6	0.1 ± 0.01	0.2 ± 0.03	0.1 ± 0.02
n-6 PUFAs	29.5 ± 0.2	27.5 ± 1.0	27.2 ± 0.9
18:3n-3	ND	ND	ND
20:5n-3	0.1 ± 0.003	0.1 ± 0.02	0.1 ± 0.01
22:6n-3	2.6 ± 0.2	2.2 ± 0.2	2.5 ± 0.3
δ^13^C 22:6n-3	−28.2 ± 0.01	−13.2 ± 0.1	87.8 ± 0.8
n-3 PUFAs	2.7 ± 0.1	2.3 ± 0.1	2.6 ± 0.2

δ13C, carbon-13; MUFA, monounsaturated fatty acid; ND, not detected; SFA, saturated fatty acid.

All values are means ± SE. Diets were measured in triplicate.

### Lipid extraction and methylation

Total lipids were extracted from diets (fish, algal, and ^13^C enriched-DHA) and tissues (brain and liver) by methodologies adapted from Folch *et al.* (11). Eppendorf tubes were used to collect the tissues after sacrifice, which were put on dry ice until stored at −80°C. Brain and liver tissue (∼70 and 100 mg, respectively) were pulverized by a tissue pulverizer (Cole-Parmer; item no. RK-36903-05) in the presence of liquid nitrogen. For the lipid extraction, total brain and liver lipids were homogenized in a 2:1 chloroform:methanol solution (v/v) with a known mass of docosatrienoic acid ethyl ester internal standard (22:3n3; NuChek Prep, Inc) for fatty acid quantification. All tissues were left exposed to extraction solvents at room temperature for 48 h. Following the 48 h period, 1.75 ml of 0.88% aqueous KCl was added, after which the tubes were inverted twice to induce separation of lipids and protein. The samples were then centrifuged at 500 *g* for 10 min, and the lower phase containing dissolved lipids (chloroform phase) were collected into a clean glass test tube. Lipids were extracted from plasma using methods described previously for harvested tissue; however, samples were immediately vortexed without a 48 h extraction.

All samples were transesterified using methods adapted from Morrison and Smith (12). A portion of the total dissolved lipids of brain, liver, and 100% of the dissolved lipids of plasma were dried down under nitrogen, and 300 μl of hexane and 1 ml of 14% BF_3_ methanol were added. The samples were vortexed and incubated in an oven at 100°C for 1 h. After samples were cooled to room temperature, 1 ml of milliQ H_2_O and 1 ml of hexane was added, and the samples were vortexed and centrifuged to induce separation. The top hexane layer containing the fatty acid methyl esters (FAMEs) was transferred into clean culture tubes and dried down under nitrogen. Culture tubes were reconstituted into 100 μl of heptane and transferred into GC vials with inserts.

### FAME quantification

Fatty acid methyl esters were quantified via GC-FID using a Varian 430 GC-FID (Scion) as previously described by our group (13). However, a DB-FFAP 30 m × 0.25 mm i.d. × 0.25 μm film thickness, nitroterephthalic acid modified, polyethylene glycol, capillary column was used (Agilent; item no. 122-3232). The column oven program was initially set at 50°C for 1 min, increased at a rate of 30°C/min to 130°C, increased at 10°C/min to 175°C, increased at 5°C/min to 230°C, and held for 9.5 min, and finally increased to 240°C at a rate of 50°C/min and held for 11.13 min, totaling 40 min. Chromatogram peaks were identified by comparing the retention time to the GLC-462 external reference standard (NuChek Prep, Inc) and quantified by comparing the peak area to that of the internal standard. After GC-FID quantification, recapped vials were then stored at−80°C until GC/C/IRMS analysis.

### Carbon isotope ratio analysis

The brain, liver, and plasma*δ*^13^C of FAMEs were determined by GC/C/IRMS. FAMEs (1 μl) were injected onto a 100 m, 0.25 mm i.d, 0.20 μm d_f_; Supelco SP-2560 (by Sigma-Aldrich) capillary column in a Thermo Scientific Trace 1310 GC interfaced to a MAT 253 IRMS (Thermo Finnigan MAT, Bremen, Germany) via a GC Iso Link II combustion interface (Thermo Scientific) using a TriPlus RSH autosampler (Thermo Fisher Scientific). Complete separation of DHA was achieved with the following program: initial temperature of 60°C with an immediate ramp of 15°C/min to 180°C with no hold, followed by a 1.5°C/min ramp to 240°C with an 18 min hold for a total run time of 66 min. The carrier flow rate was set to 1.2 ml/min, yielding baseline resolutions of analyte peaks of interest. As a result of helium carrier gas, the GC effluent was swept to a Thermo Fisher Scientific GC Iso Link II combustion interface at 1,000°C, with nickel and copper catalysts, and the MAT 253 IRMS (Thermo Fisher Scientific) was interfaced via a ConFlo IV (Thermo Fisher Scientific) continuous-flow interface. Prior to entering the IRMS ion source (Dupont, Wilmington, DE), the CO_2_ gas produced by quantitative combustion of isolated analytes was dried by flowing gas through a Nafion dryer. With each batch of samples, heptadecanoic acid and methyl heptadecanoate were analyzed by elemental analysis to determine the carbon isotopic ratio of methyl groups esterified to generate FAMEs from the total lipid extract. The solvent was evaporated overnight at room temperature from aliquots (150 μg of total material) transferred to thin-walled tin capsules (Elemental Microanalysis, Okehampton, UK). Capsules were introduced into the Flash 2000 Elemental Analyzer (Thermo Fisher Scientific) using a Zero Blank autosampler (Costech Analytical Technologies, Inc, Valencia, CA). In the oxidation and reduction reactors, temperatures were maintained at 1,020°C and 650°C, respectively. Using a ConFlo IV continuous-flow interface (Thermo Fisher Scientific), CO_2_ gas was introduced to the Delta V Plus IRMS similarly to the GC/C/IRMS setup.

### Carbon isotope ratio normalization and methyl corrections

Using multipoint linear normalization similar to previously described methods (13, 14), all carbon isotope ratios were normalized with consensus-validated 20-carbon FAME reference material USGS70, USGS71, and USGS72 (Reston Stable Isotope Laboratory). In USGS70, USGS71, and USGS72, consensus-derived carbon isotope ratios are −30.53, −10.50, and −1.54, respectively and are expressed relative to Vienna Peedee Belemnite on a scale normalized to primary reference materials NBS 19 and LSVE (15). Under similar analytical conditions to the analyzed samples, reference materials were injected twice throughout each batch of samples analyzed by GC/C/IRMS and elemental analysis/IRMS. Based on the measured and accepted ^13^C values of reference materials, linear regression lines were used to generate normalizing equations for each batch of samples. *R*^*2*^ values for all equations were 0.9998. CO_2_ gas produced through the combustion of sample material or specific organic analytes is measured by IRMS, which cannot distinguish between derivatized carbon units added to fatty acids during transmethylation. By using the following mass balance equation, methyl corrections were performed to account for the contribution of derivatized carbon units to measured carbon isotope ratios, in order to report true values.

(Eq. 1) $$n_{FAME}\delta^{13}C_{FAME}=\delta^{13}C_{ME}+n_{FA}\delta^{13}C_{FA}$$

δ^13^C is defined as the isotope ratio of ^13^C to ^12^C. The subscripts FAME, ME, and FA are the measured carbon isotope ratios of FAMEs, methyl groups, and fatty acids, respectively, where *n* refers to the number of moles of carbon in the corresponding FAMEs and fatty acids. nFAME and nFA are equal to 23 and 22, respectively, for methyl corrections of measured DHA carbon isotope ratios. In each batch of samples, carbon isotope ratios of the methyl group were determined using methylated and free heptadecanoic acid standards (Nu-Chek Prep, Inc). The carbon isotope signature of the methyl group was −40.5 mUr.

### Statistics and calculations

All reported data are expressed as means ± SEs. The sample size ranged from three to four mice per time point throughout the study. A two-way ANOVA was used to compare brain, liver, and plasma DHA concentrations in mice after the diet switch using the interaction of time and diet group at *P* < 0.05. When an interaction was observed, a one-way ANOVA was performed, with significance determined at *P* < 0.05. Statistical significance for half-life calculations was determined by nonoverlapping 95% CIs.

Tissue δ^13^C-DHA signatures from mice following the diet switch were plotted over time and modeled with a one-phase decay function to generate a curve of best fit:

(Eq. 2) $$y={\left(y_{0}-plateau\right)}^{-kx}+plateau$$

$y_{0}$ defines the best-fit curve at time 0, plateau defines the value of the best-fit curve at an infinite time, and *k* defines the decay constant. Incorporation half-lives for tissue δ^13^C-DHA signatures were determined from the best-fit curves using the following formula:

(Eq. 3) $$t_{1/2}=\frac{\ln\left(2\right)}{k}$$

Based on a one-phase decay function, *k* is the decay constant. The following formula was used to calculate the rate of loss of DHA from the tissue DHA pool, *J*_out_ (μmol/g brain/day) (16, 17, 18):

(Eq. 4) $$J_{out}=\frac{0.693C_{FA}}{t_{1/2}}$$

*C*_*FA*_ is the baseline DHA concentration from the given tissue lipid pool, and *t*_1/2_ is the corresponding experimentally derived half-life.

To approximate the net incorporation rate of DHA, the rate of loss of DHA from the DHA tissue pool, *J*_out_, must be assumed to be equal when the net incorporation rate of DHA and the net loss rate of DHA are at steady state (4).

GraphPad Prism, version 9 (GraphPad Software, San Diego, CA) was used to perform the two-way ANOVA, one-way ANOVA, and one-phase exponential decay function tests.

## RESULTS

### Fatty acid concentration of DHA in brain, liver, and plasma tissues after diet switch

**Fig. 2:**
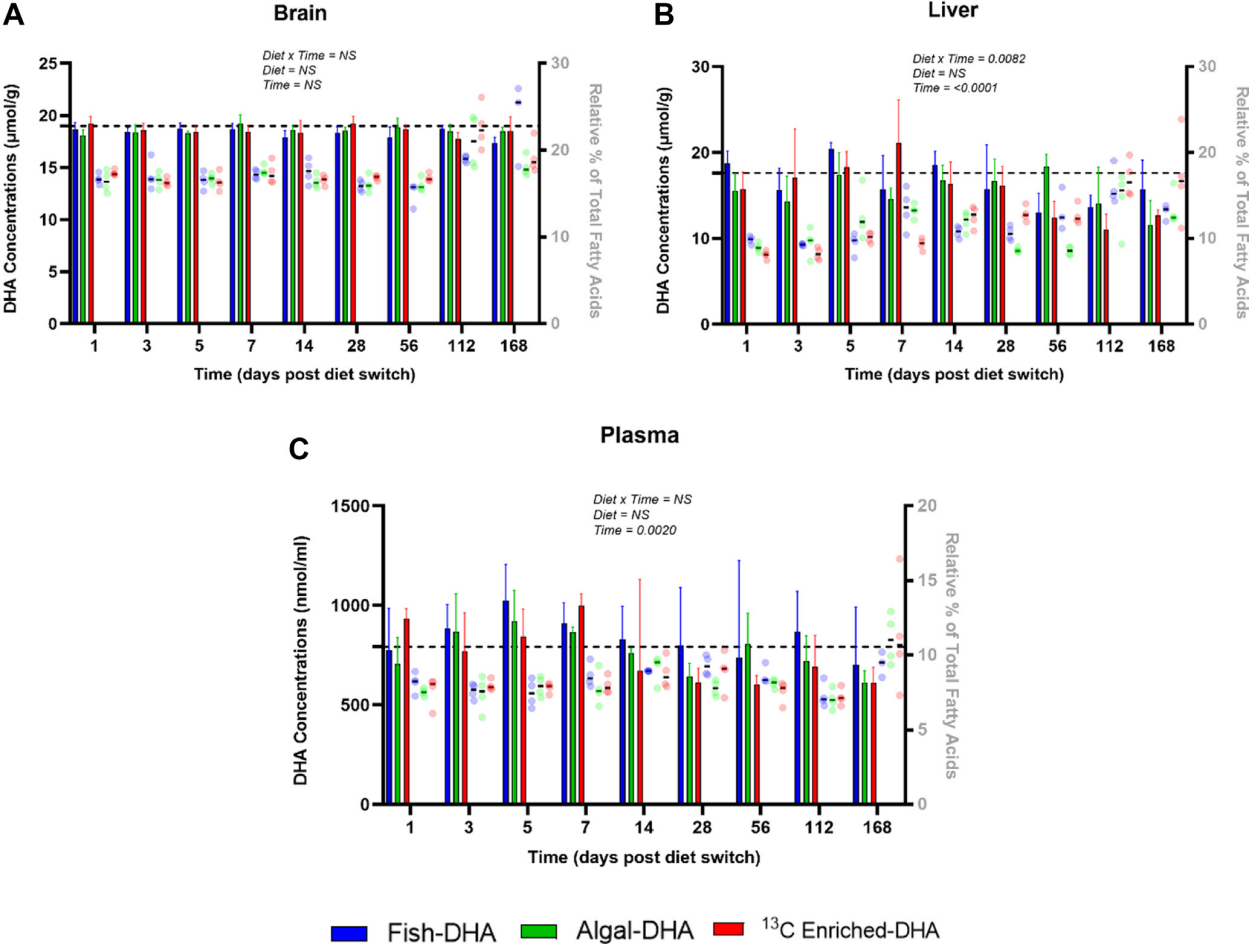
Brain (A), liver (B), and plasma (C) DHA concentrations (bars) and DHA relative percent of total fatty acids (dots) following the diet switch from mice equilibrated to the control (fish-DHA) diet. Data are shown as means ± SEs and were compared by two-way ANOVA for the interaction of time and diet (*n* = 3–4 per time point). *P* < 0.05 was considered statistically significant. Baseline DHA concentrations are represented by the dashed line with the black band. NS, not significant.

Brain, liver, and plasma tissues were collected from mice consuming the fish, algal, and ^13^C enriched-DHA diets throughout the time course of 168 days (Fig. 1). DHA concentrations and DHA relative percent of total fatty acids of brain, liver, and plasma are presented in Fig. 2A, B, C, respectively. Brain, liver, and plasma fatty acid concentrations of mice fed DHA diets at each time point are displayed in supplemental Tables S1–S3, respectively.

After the diet switch, throughout the time course, brain DHA concentrations showed no significant interaction of diet and time, and neither diet nor time affected DHA concentrations (Fig. 2A). The DHA baseline concentration for the brain was ∼19 μmol/g and had a range between ∼17 and 19 μmol/g. A significant time by diet interaction (*P* = 0.0082) was observed for liver DHA concentrations (Fig. 2B). The DHA liver concentrations had a range between ∼11 and 21 μmol/g, with a baseline DHA value of ∼18 μmol/g. Plasma DHA concentrations showed no interaction of diet or time, although a significant effect of time was observed (*P* = 0.0020) (Fig. 2C). The DHA plasma baseline concentration had a value of 792 nmol/ml and had a range between 600 and 1,025 nmol/ml.

### Carbon isotopic analysis for brain, liver, and plasma tissues

In control mice equilibrated to the fish-DHA diet over 3 months, we were able to establish a stable DHA isotopic signature of −25.3 ± 0.10, −27.4 ± 0.10, and −28.3 ± 0.50 mUr for the brain, liver, and plasma, respectively.

**Fig. 3:**
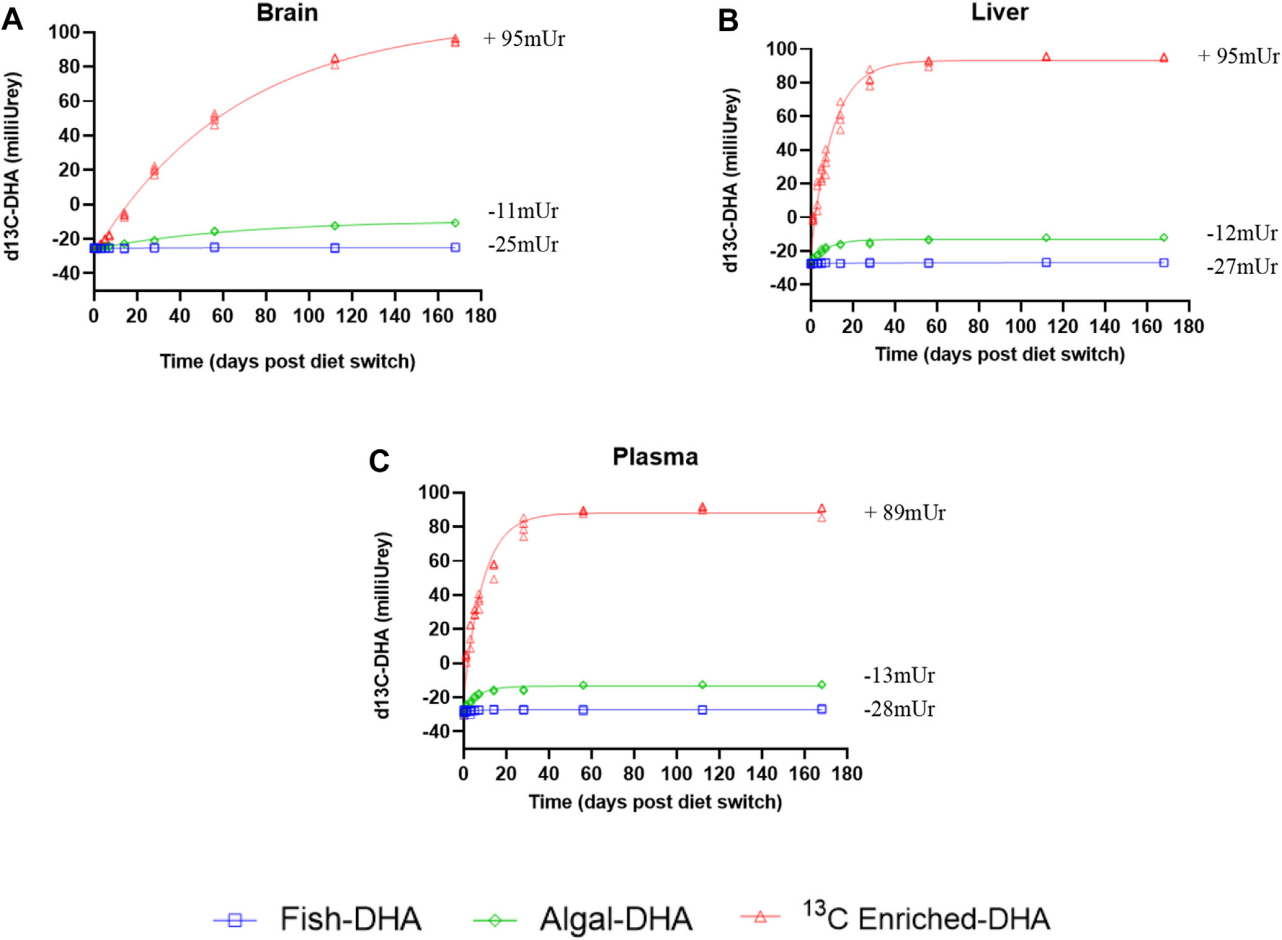
Change in brain (A), liver (B), and plasma (C) δ^13^C _DHA_ signatures following the diet switch from mice equilibrated to the control (fish-DHA) diet. Data are shown as individual data points and modeled with a one-phase decay function (*n* = 3–4 mice per time point).

**Fig. 4:**
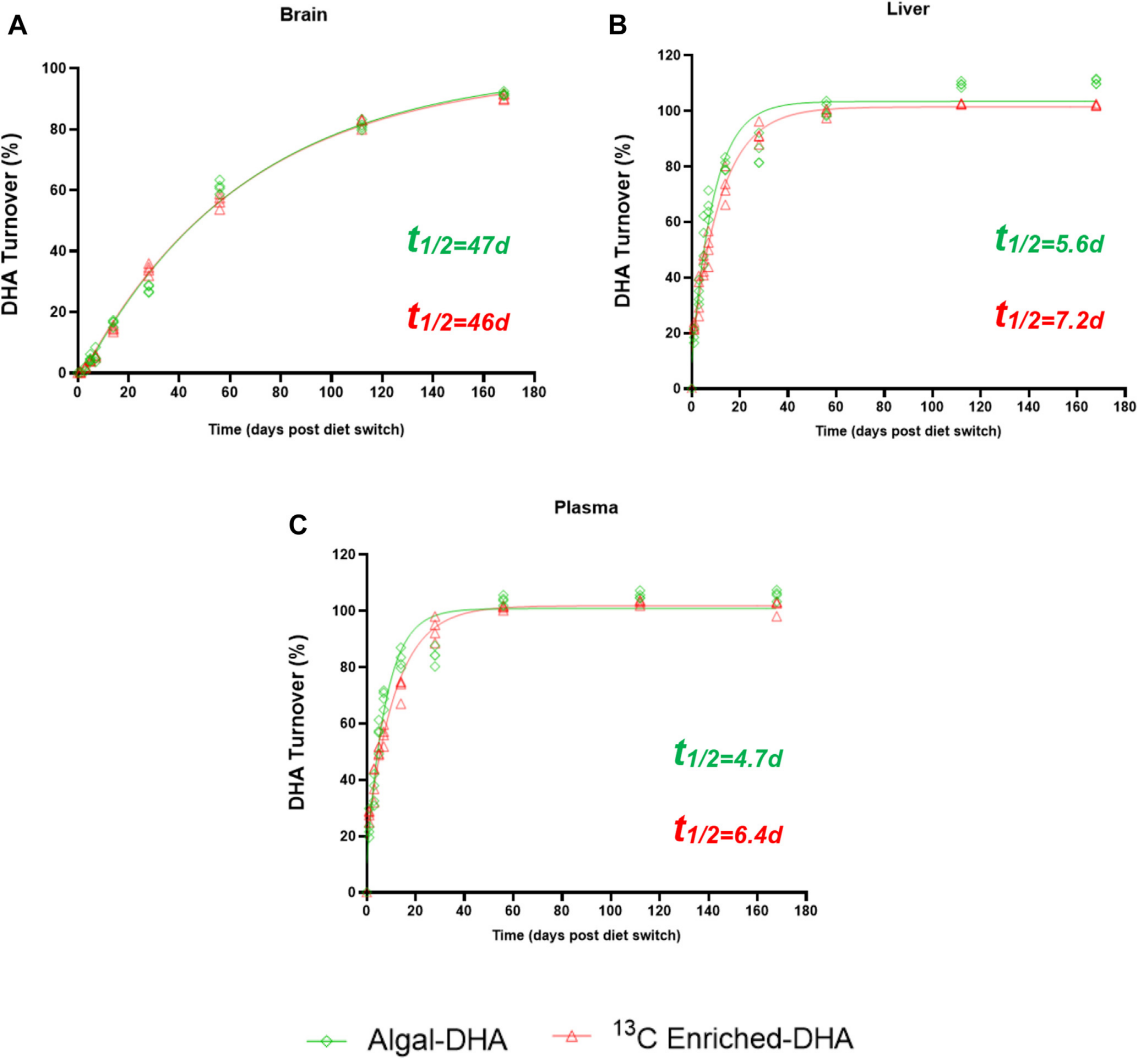
Percent DHA turnovers in brain (A), liver (B), and plasma (C) tissues following the diet switch from mice equilibrated to the control (fish-DHA) diet. Data are shown as individual data points (*n* = 3–4 mice per time point). *t*_1/2_, half-life.

Changes in brain, liver, and plasma δ^13^C-DHA signatures following the diet switch are displayed in Fig. 3, and their respective percent DHA turnovers are displayed in Fig. 4. Over the 168-day period, brain, liver, and plasma DHA signatures were measured and fitted to a one-phase decay function to model DHA tissue turnover.

Kinetic parameters from best-fit curves of δ^13^C-DHA signatures in response to the diet switch after equilibration of the fish-DHA (control) diet are displayed in Table 2. For the brain, liver, and plasma, incorporation half-lives were determined from mice equilibrated on the control DHA diet using Equation 3 (see Statistics and calculations section). The brain DHA half-life was 46.9 days (95% CI, 41.98–52.92) in mice fed the algal-DHA diet and 46.2 days (95% CI, 42.92–49.94) in mice fed the ^13^C enriched-DHA diet. The liver DHA half-life was 5.6 days (95% CI, 4.64–6.81) in mice fed the algal-DHA diet and 7.2 days (95% CI, 6.32–8.26) in mice fed the ^13^C enriched-DHA diet. The plasma DHA half-life was 4.7 days (95% CI, 3.91–5.75) in mice fed the algal-DHA diet and 6.4 days (95% CI, 5.40–7.58) in mice fed the ^13^C enriched-DHA diet (Table 2). Based on overlapping 95% CIs, there were no statistical differences in brain, liver, and plasma DHA half-lives between diet switch groups. Brain DHA incorporation half-lives were ∼6- to 8-fold longer than the liver half-lives (6-fold longer in mice fed the ^13^C enriched-DHA diet and 8-fold longer in mice fed the algal-DHA diet). Brain DHA incorporation half-lives were 7-10-fold longer than the plasma half-lives (7-fold longer in mice fed the ^13^C enriched-DHA diet and 10-fold longer in mice fed the algal-DHA diet). The liver and plasma incorporation half-lives were similar; the liver half-life was 1.2-fold longer than the plasma half-life in mice fed the algal-DHA diet, and the liver half-life was 1.1-fold longer than the plasma half-life in mice fed the ^13^C enriched-DHA diet. The brain, liver, and plasma DHA incorporation half-lives yielded similar results in mice switched from the control diet on to its respective algal versus ^13^C enriched-DHA diet. Half-lives and kinetic parameters could not be established for the control mice maintained on the fish-DHA diet because δ^13^C-DHA remained unchanged throughout the time course. This group was not adequately modeled by a one-phase decay function.

**Table 2:** Kinetic parameters from best-fit curves of δ^13^C-DHA signatures in response to the diet switch after equilibration of the fish (control) diet

Tissue	Diet	y_0_, mUr	Plateau, mUr	k, days	*t*_1/2_, days (95% CI)	*J*_out_
Brain	Algal-DHA	−25.78	−9.14	0.015	46.94 (41.98–52.92)	0.28 μmol/g brain/day
	^13^C Enriched-DHA	−28.36	107.80	0.015	46.22 (42.92–49.94)	0.28 μmol/g brain/day
Liver	Algal-DHA	−26.84	−13.04	0.124	5.59 (4.64–6.81)	2.23 μmol/g liver/day
	^13^C Enriched-DHA	−20.02	93.25	0.096	7.22 (6.32–8.26)	1.73 μmol/g liver/day
Plasma	Algal-DHA	−27.78	−13.35	0.147	4.72 (3.91–5.75)	116.28 nmol/ml plasma/day
	^13^C Enriched-DHA	−18.84	88.12	0.109	6.38 (5.40–7.58)	86.03 nmol/ml plasma/day

*J*_out_, synthesis rate; k, decay constant; Plateau, value of the best-fit curve at an infinite time; *t*_1/2_, half-life; y_0_, best-fit curve at time 0.

A one-phase decay function was utilized to generate kinetic parameters based on best-fit curves generated through δ^13^C DHA signatures. Significance for half-lives was determined by nonoverlapping 95% CIs.

### The rate of loss of DHA (*J*_out_)

Because DHA concentrations in the brain were stable, and relatively stable in the liver, and plasma tissues, total lipid *J*_out_ values were calculated for brain, liver, and plasma (Table 2). Estimates of the daily synthesis rates or daily rate of loss (*J*_*out*_) are determined by using Equation 4 (see Statistics and calculations section). The rate of DHA out of a tissue is approximately the rate of incorporation of DHA into a tissue, given that the DHA tissue pool sizes remain constant. The rate of loss of brain DHA was 6-8-fold slower than the rate of loss of liver DHA (6-fold slower in mice fed the ^13^C enriched-DHA diet and 8-fold slower in the mice fed the algal-DHA diet). The rate of loss of brain DHA in the algal and ^13^C enriched-DHA diets was the same; 0.28 μmol/g brain/day. The rate of loss of liver DHA in the algal-DHA fed mice was approximately 22% faster than the mice on the ^13^C enriched-DHA diet, and the rate of loss of plasma DHA in the algal-DHA fed mice was approximately 26% faster than the mice on the ^13^C enriched-DHA diet; however, because of how rate of loss was calculated, these putative differences were not analyzed statistically.

## DISCUSSION

By combining a diet switch study with natural abundance CSIA, previously used by Lacombe *et al.*, it has been shown that changes in tissue δ^13^C signatures in response to the altered δ^13^C signature of the diets can be used to estimate brain DHA half-lives (4). By equilibrating the brain δ^13^C DHA signature with that of the dietary n-3 PUFA (either ALA or DHA) and switching mice to a diet providing n-3 PUFA with a different carbon isotopic signature, changes in brain δ^13^C DHA signatures were modeled as a function of time, which allowed for the determination of brain DHA half-lives and turnover. Although the main outcome of the study by Lacombe *et al.* was to measure brain DHA half-lives, this approach was limited first by the selection of time points that missed the window of DHA turnover in peripheral tissues. While modeling DHA tissue turnover in the liver, Lacombe *et al.* showed a plateau before his first time point of 7 days suggesting that DHA had already turned over within the first week. Therefore, the current study took advantage of using earlier time points (day 1, 3, and 5) to catch the turnover of tissues other than the brain, such as liver, and plasma (Fig. 1). Second, the lack of steady-state DHA tissue pool sizes in the study by Lacombe *et al.*' peripheral tissues limited accurate calculations of turnover. The net rate of DHA incorporation approximates the net rate of DHA loss assuming that the DHA tissue pool sizes are at steady state (19). According to the limitations of Lacombe *et al.*, the large accretion of DHA in liver and adipose pools most likely contributed to an underestimation of exact DHA half-lives in these peripheral tissues (4). This was due to using a diet switch containing two n-3 PUFA (ALA and DHA) from two sources resulting in variable DHA pool sizes. When switching from DHA to ALA, peripheral DHA stores of high δ^13^C remain high post diet switch and continue to contribute to a higher δ^13^C for days. Because peripheral DHA stores were low at the start of Lacombe *et al.*'s diet switch from ALA to DHA, DHA levels in tissues increased rapidly. Therefore, in the current study, by manipulating the δ^13^C isotopic signature of only DHA using two different sources; fish-DHA, and an isotopically enriched algal-DHA source, this helped maintain stable DHA pools to better assess DHA tissue turnover. The isotopically enriched DHA algal source (*Crypthecodinium cohnii*) is fed a corn-based carbohydrate, which is a C4 plant making the algal-DHA diet more enriched in δ^13^C compared with the fish-DHA diet.

The δ^13^C signatures for the fish-DHA and algal-DHA diets were determined to be −28.2 and −13.2 mUr, respectively (Table 1). By equilibrating the tissue δ^13^C DHA signature with that of one dietary n-3 PUFA (DHA) and switching mice to a diet providing the same n-3 PUFA, with a different δ^13^C DHA signature (algal-DHA), changes in tissue δ^13^C DHA signatures were modeled as a function of time, allowing for the determination of brain, liver, and plasma DHA half-lives and turnover. Therefore, by using earlier time points, and substituting DHA sources by keeping the n-3 PUFA the same, we were able to better calculate DHA turnover in the liver and plasma.

In mice equilibrated to the control diet and switched on to the algal-DHA diet, the brain half-life was determined to be 47 days (Table 2). As we expected, these values yielded consistent results compared with previously published studies such as Lacombe *et al.*, where the brain half-life was determined to be approximately 33–39 days. Despite these consistent results, some methodological differences could have contributed to the shorter half-life calculated in the study by Lacombe *et al.* such as diets. The faster turnover of 33–39 days of Lacombe *et al.* could have been due to mice starting on the ALA diet (initially low DHA stores), and then by switching to the DHA diet resulted in a pool of DHA available for brain uptake that was not diluted by contributions from those peripheral tissue stores. In the current study, we switched from a DHA-to-DHA diet where peripheral tissues are already high and at steady state. This suggests that brain DHA turnover by Lacombe *et al.* could have been faster because of the influx of DHA relative to the availability of DHA from peripheral tissues.

Studies measuring the loss of brain radioactivity in rats by using radioactive tracers (via intracerebroventricular injection) to determine brain DHA half-lives also showed consistent results that were between 30 and 64 days (16, 17, 20). The half-life results of brain DHA by DeMar *et al.* was 33 days, and the *J*_out_ calculation was 0.26 μmol/g brain/day (16), half-life results by Lin *et al.* were 33 days, and the *J*_out_ value was 0.13 μmol/g (17), whereas the results of Chen *et al.* yielded a half-life of 64 days, and a *J*_out_ of 0.065 μmol/g brain/day (20). DeMar *et al.*, Lin *et al.*, and Chen *et al.* determined half-lives and *J*_out_ values that are broadly similar to each other despite methodological differences. Slight differences such as the strain of the rat used could affect the half-life calculations. Chen *et al.* used Sprague-Dawley rats that are an outbred stock with genetic variation that may affect fatty acid metabolism. Another factor that may affect the half-life are the diets used in each study. Although ALA remained similar between adequate diets in all three studies, the study diet of Chen *et al.* used had considerably higher amounts of LA (54%) compared with ALA (5%), which may influence DHA levels. There is competition between n-6 and n-3 PUFA for the desaturation enzymes, D5D and D6D, and elongase enzymes, elongase 2 and elongase 5 (6). High intakes of LA may interfere with the desaturation and elongation of the n-3 PUFA in the liver, thereby affecting DHA levels in the brain. Furthermore, the n-3 PUFA-deprived rats had a 2-fold increase in loss of half-life in total PL and a 77% decrease in the *J*_out_ values compared with the n-3 PUFA adequate rats. This shows that there are mechanisms to conserve DHA within the brain and that these mechanisms are upregulated during deprivation (16).

Our *J*_out_ calculation yielded 0.28 μmol/g brain/day (Table 2), which was similar to Lacombe’s *J*_out_ calculation of ∼0.32–0.38 μmol/g brain/day, and also broadly similar to *J*_out_ calculations for Lin *et al.* and DeMar *et al.* (0.13–0.26 μmol/g). In conclusion, the results reveal that brain half-lives and *J*_out_ values were consistent with those previously determined from the diet switch study of Lacombe *et al.* and from radiolabeled kinetic modeling studies.

In line with Lacombe *et al.*’s liver DHA half-life estimates of 2.5–17 days, from the ALA to DHA and DHA to ALA diet switch, respectively (4) the liver half-life in the current study was calculated to be 5.6 and 7.2 days in mice equilibrated to the control diet and switched onto the algal-DHA diet and ^13^C enriched-DHA, respectively (Table 2). This result suggests that by incorporating time points within the first week, we were able to measure DHA turnover more accurately in the liver tissue. Moreover, we were able to pinpoint this result because of maintenance of stable DHA liver pool sizes allowing us to make a proper turnover/half-life calculation. Liver DHA concentrations at baseline started at 18 μmol/g and remained relatively stable throughout (Fig. 2B). The rate of loss (*J*_out_) of liver DHA in mice switched to the algal-DHA diet, which was 2.23 μmol/g liver/day (Table 2), was calculated because we were able to maintain relatively stable DHA pool sizes in the liver. In comparison with the results of Lacombe *et al.*, his estimates of liver DHA half-lives could have been the result of liver DHA concentrations experiencing a large increase or decrease from baseline depending on the background-equilibrated diet and consequently was unable to perform *J*_out_ calculations. By using a diet switch study in conjunction with CSIA while improving methodological limitations, we were able to estimate DHA turnover more accurately in the liver.

The second part of this current study was to validate the utility of a ^13^C enriched-DHA source (uniformly labeled ^13^C DHA) in diet switch studies by adding one more diet source of DHA to the study design, which we referred to as the ^13^C enriched-DHA diet (Fig. 1). We purchased uniformly labeled carbon-13 DHA (Cambridge Isotope Laboratories, Inc) and formulated the diet by enriching a fish-DHA source with 0.25% of uniformly labeled ^13^C DHA to increase the δ^13^C to +87.8 mUr allowing the signatures of all three diets to be different. Therefore, at the same time, one group of mice was switched on to the algal-DHA diet, and another group was switched on to the uniformly labeled DHA (^13^C enriched-DHA diet) at a +87.8 mUr signature (Fig. 1).

In mice equilibrated to the control diet and switched onto the ^13^C enriched-DHA diet, the brain half-life was determined to be 46 days, and the *J*_out_ was determined to be 0.28 μmol/g brain/day almost identical to the rate obtained upon switching to the algal-DHA diet (Table 2). Similarly, the liver half-life of 7.2 days and the *J*_out_ of 1.73 μmol/g liver/day in the mice switched onto the ^13^C enriched-DHA diet, yielded similar rates to mice switched onto the algal-DHA diet. Therefore, this validated the use of the ^13^C enrichment of DHA in diets to measure brain DHA turnover and half-lives. These findings have implications for future studies, such as measuring the metabolism of fatty acids such as ALA or palmitic acid, which do not have naturally abundant high carbon-13 sources in the food supply. In addition, we can measure the effects of diet, genetics, or stress on brain fatty acid metabolism using this enrichment method. Studies can also be conducted by measuring the turnover of fatty acids in the blood, such as DHA, by providing humans with different sources of DHA.

In addition, we were able to calculate the DHA half-life in the plasma. The plasma DHA half-life was 4.7 days in mice fed the algal-DHA diet and 6.4 days in mice fed the ^13^C enriched-DHA diet. The 95% CIs in plasma half-lives overlapped, resulting in no statistical significance between each diet switch. Interestingly, the plasma and liver half-lives showed similar results indicating a relationship between fatty acid turnover in the liver and plasma.

One limitation of this experiment is that mice were not fasted before being sacrificed; therefore, some of the carbon isotope ratio measurements in tissues with shorter half-lives may have been affected by the postprandial circulating TAG in their blood. There were some variations in carbon isotopic ratios in the liver tissues on day 3, 5, 7, and 14. In future studies investigating liver fatty acid turnover, animals should be fasted before sample collection to limit variations in carbon isotope ratios resulting from recently consumed diets. A second limitation was that mice were not weighed, and food intake was not tracked throughout the time course. Variations in carbon isotopic ratios and concentration in the liver could have been due to the amount of food the mice were consuming. This could be tracked in the future by determining food intake and/or mass of the mice. Another limitation of the study was that my control fish-DHA diet and algal-DHA diet were both ethyl ester DHA sources, whereas the ^13^C enriched-DHA diet was a methyl ester DHA source. Although there have been no studies done to compare ethyl ester versus methyl ester absorption, ethyl ester absorption has been shown in various studies to be absorbed slower in the body compared with TAGs because of the fatty acid-ethanol bond in the ethyl ester, which is up to 50 times more resistant to pancreatic lipase as compared with the hydrolysis of the glycerol backbone in TAGs (21, 22). Because of these potential differences, the turnover of both algal and ^13^C enriched groups could have been slightly different when mice were switched onto their respective diets.

In summary, our study has shown that by improving limitations in previous models (time point selections and unstable DHA pool sizes) we were able to better determine DHA turnover in the brain and for the first time, more accurately calculate turnover of DHA in tissues other than the brain, such as liver and plasma. Also, because CSIA is noninvasive, future studies could use CSIA to study human blood. It was also shown, for the first time, by utilizing an enriched DHA source (unnaturally high δ^13^C), we were able to validate its utility in diet switch studies. Validation of the uniformly ^13^C labeled DHA allows for the use of molecules that lack natural variations in δ^13^C such as ALA.

## Data availability

All datasets generated during and/or analyzed during the current study are available from the corresponding author on reasonable request.

## Supplemental data

This article contains supplemental data.
