# Supplementary material for: Novel 13C enrichment technique reveals early turnover of DHA in peripheral tissues
Source: J Lipid Res. 2023 Mar 21;64(5):100357. doi: 10.1016/j.jlr.2023.100357 (PMC10154972; doi:10.1016/j.jlr.2023.100357)
Supplement: Supporting information [file mmc1.docx]

**SUPPLEMENTAL INFORMATION:**

**Novel ^13^C enrichment technique reveals early turnover of DHA in peripheral tissues**

Brinley J. Klievik^1^, Adam H. Metherel^1^, Giulia Cisbani^1^, Rodrigo Valenzuela^1^, and Richard P. Bazinet*,^1^

^1^Department of Nutritional Sciences, Temerty Faculty of Medicine, University of Toronto, 1 King’s College Circle, Toronto, Ontario, Canada, M5S 1A8

| **Supplemental Table 1A. Brain fatty-acid concentrations of mice fed fish-DHA diet (control) at baseline** | |
| --- | --- |
|  | Concentration of fatty acids (umol/g) |
| Fatty acid | Fish-DHA (n=6) |
| 10:0 | 0 ± 0 |
| 12:0 | 0.03 ± 0 |
| 14:0 | 0.85 ± 0.13 |
| 16:0 | 32.31 ± 1.28 |
| 17:0 | 0.23 ± 0.01 |
| 18:0 | 25.88 ± 0.81 |
| 20:0 | 0.46 ± 0.03 |
| 22:0 | 0.28 ± 0.02 |
| 23:0 | 0.08 ± 0.01 |
| 24:0 | 0.28 ± 0.02 |
| ***SFAs*** | *60.39 ± 0.14* |
| 14:1 | 0.28 ± 0.06 |
| 16:1n-7 | 0.85 ± 0.04 |
| 16:1n-9 | 0.81 ± 0.12 |
| 18:1n-7 | 4.93 ± 0.16 |
| 18:1n-9 | 21.96 ± 0.82 |
| 20:1n-9 | 2.76 ± 0.19 |
| 22:1n-9 | 0.25 ± 0.02 |
| 24:1n-9 | 0.46 ± 0.05 |
| ***MUFAs*** | *32.3 ± 0* |
| 18:2n-6 | 0.93 ± 0.03 |
| 18:3n-6 | 0.02 ± 0 |
| 20:2n-6 | 0.27 ± 0.02 |
| 20:3n-6 | 0.7 ± 0.02 |
| 20:4n-6 | 10.63 ± 0.29 |
| 22:2n-6 | 0.05 ± 0.01 |
| 22:4n-6 | 2.76 ± 0.07 |
| 22:5n-6 | 0.19 ± 0 |
| ***n-6 PUFAs*** | *15.55 ± 0.03* |
| 18:3n-3 | 0 ± 0 |
| 20:5n-3 | 0.08 ± 0 |
| 22:5n-3 | 0.19 ± 0 |
| 22:6n-3 | 18.98 ± 0.3 |
| ***n-3 PUFAs*** | *19.25 ± 0.07* |

| **Supplemental Table 1B. Brain fatty-acid concentrations of mice fed fish, algal and C^13^ enriched-DHA diets at day 1** | | | |
| --- | --- | --- | --- |
|  | Concentration of fatty acids (umol/g) | | |
| Fatty acid | Fish-DHA (n=4) | Algal-DHA (n=4) | C^13^ Enriched-DHA (n=4) |
| 10:0 | 0.01 ± 0 | 0.01 ± 0 | 0.01 ± 0 |
| 12:0 | 0.04 ± 0 | 0.05 ± 0.01 | 0.05 ± 0.01 |
| 14:0 | 0.89 ± 0.23 | 1.12 ± 0.09 | 0.26 ± 0.02 |
| 16:0 | 31.75 ± 2.04 | 31.55 ± 2.01 | 32.9 ± 0.73 |
| 17:0 | 0.23 ± 0.01 | 0.22 ± 0.01 | 0.23 ± 0.01 |
| 18:0 | 25.63 ± 0.78 | 25.02 ± 0.97 | 26.02 ± 0.56 |
| 20:0 | 0.49 ± 0.03 | 0.53 ± 0.08 | 0.38 ± 0.03 |
| 22:0 | 0.31 ± 0.02 | 0.32 ± 0.04 | 0.24 ± 0.02 |
| 23:0 | 0.09 ± 0.01 | 0.09 ± 0.01 | 0.07 ± 0.01 |
| 24:0 | 0.34 ± 0.04 | 0.34 ± 0.04 | 0.26 ± 0.02 |
| ***SFAs*** | *59.77 ± 0.21* | *59.23 ± 0.21* | *60.41 ± 0.08* |
| 14:1 | 0.3 ± 0.1 | 0.39 ± 0.03 | 0.01 ± 0 |
| 16:1n-7 | 0.88 ± 0.1 | 0.86 ± 0.06 | 1.01 ± 0.05 |
| 16:1n-9 | 0.84 ± 0.21 | 1.06 ± 0.08 | 0.23 ± 0 |
| 18:1n-7 | 4.98 ± 0.18 | 4.92 ± 0.28 | 4.92 ± 0.17 |
| 18:1n-9 | 22.63 ± 0.5 | 22.5 ± 1.39 | 21.56 ± 0.63 |
| 20:1n-9 | 2.97 ± 0.15 | 3.13 ± 0.45 | 2.37 ± 0.18 |
| 22:1n-9 | 0.27 ± 0.02 | 0.29 ± 0.03 | 0.21 ± 0.01 |
| 24:1n-9 | 0.53 ± 0.05 | 0.52 ± 0.05 | 0.36 ± 0.02 |
| ***MUFAs*** | *33.39 ± 0.05* | *33.66 ± 0.16* | *30.65 ± 0.08* |
| 18:2n-6 | 0.91 ± 0.06 | 0.91 ± 0.07 | 0.95 ± 0.07 |
| 18:3n-6 | 0.01 ± 0 | 0.01 ± 0 | 0.01 ± 0 |
| 20:2n-6 | 0.2 ± 0.04 | 0.18 ± 0.03 | 0.23 ± 0.03 |
| 20:3n-6 | 0.67 ± 0.01 | 0.71 ± 0.03 | 0.69 ± 0.02 |
| 20:4n-6 | 10.5 ± 0.2 | 10.15 ± 0.38 | 10.65 ± 0.23 |
| 22:2n-6 | 0.03 ± 0.01 | 0.03 ± 0.01 | 0.03 ± 0.01 |
| 22:4n-6 | 2.83 ± 0.06 | 2.8 ± 0.04 | 2.58 ± 0.07 |
| 22:5n-6 | 0.21 ± 0 | 0.18 ± 0.02 | 0.19 ± 0.01 |
| ***n-6 PUFAs*** | *15.36 ± 0.02* | *14.97 ± 0.04* | *15.31 ± 0.03* |
| 18:3n-3 | 0.01 ± 0.01 | 0 ± 0 | 0.02 ± 0 |
| 20:5n-3 | 0.08 ± 0 | 0.08 ± 0 | 0.07 ± 0 |
| 22:5n-3 | 0.19 ± 0 | 0.19 ± 0.01 | 0.18 ± 0 |
| 22:6n-3 | 18.67 ± 0.32 | 18.08 ± 0.27 | 19.23 ± 0.33 |
| ***n-3 PUFAs*** | *18.94 ± 0.08* | *18.35 ± 0.07* | *19.5 ± 0.08* |

| **Supplemental Table 1C. Brain fatty-acid concentrations of mice fed fish, algal and C^13^ enriched diets at day 3** | | | |
| --- | --- | --- | --- |
|  | Concentration of fatty acids (umol/g) | | |
| Fatty acid | Fish-DHA (n=4) | Algal-DHA (n=4) | C^13^-Enriched (n=4) |
| 10:0 | 0.01 ± 0 | 0.01 ± 0 | 0 ± 0.01 |
| 12:0 | 0.03 ± 0.01 | 0.05 ± 0.02 | 0.1 ± 0.03 |
| 14:0 | 0.7 ± 0.27 | 0.42 ± 0.16 | 1.04 ± 0.25 |
| 16:0 | 30.94 ± 3.07 | 32.27 ± 1.63 | 32.81 ± 1.54 |
| 17:0 | 0.22 ± 0.02 | 0.23 ± 0.01 | 0.23 ± 0.01 |
| 18:0 | 25.17 ± 1.49 | 25.88 ± 0.89 | 25.6 ± 0.7 |
| 20:0 | 0.48 ± 0.03 | 0.5 ± 0.03 | 0.51 ± 0.02 |
| 22:0 | 0.31 ± 0.02 | 0.32 ± 0.02 | 0.31 ± 0.01 |
| 23:0 | 0.09 ± 0.01 | 0.09 ± 0 | 0.09 ± 0 |
| 24:0 | 0.33 ± 0.02 | 0.35 ± 0.03 | 0.34 ± 0.01 |
| ***SFAs*** | *58.26 ± 0.32* | *60.11 ± 0.17* | *61.04 ± 0.16* |
| 14:1 | 0.22 ± 0.12 | 0.08 ± 0.07 | 0.34 ± 0.11 |
| 16:1n-7 | 0.88 ± 0.1 | 0.96 ± 0.08 | 1.01 ± 0.06 |
| 16:1n-9 | 0.65 ± 0.25 | 0.39 ± 0.19 | 0.97 ± 0.25 |
| 18:1n-7 | 4.78 ± 0.32 | 5 ± 0.19 | 5.15 ± 0.11 |
| 18:1n-9 | 21.61 ± 1.45 | 22.61 ± 0.98 | 23.25 ± 0.38 |
| 20:1n-9 | 2.81 ± 0.22 | 2.95 ± 0.15 | 3.14 ± 0.11 |
| 22:1n-9 | 0.26 ± 0.02 | 0.26 ± 0.01 | 0.28 ± 0 |
| 24:1n-9 | 0.43 ± 0.08 | 0.5 ± 0.05 | 0.42 ± 0.08 |
| ***MUFAs*** | *31.65 ± 0.17* | *32.74 ± 0.11* | *34.56 ± 0.04* |
| 18:2n-6 | 0.83 ± 0.07 | 0.89 ± 0.05 | 1.05 ± 0.11 |
| 18:3n-6 | 0.01 ± 0 | 0.01 ± 0 | 0.01 ± 0 |
| 20:2n-6 | 0.2 ± 0.01 | 0.2 ± 0 | 0.23 ± 0.02 |
| 20:3n-6 | 0.69 ± 0.03 | 0.68 ± 0.02 | 0.75 ± 0.02 |
| 20:4n-6 | 10.33 ± 0.33 | 10.51 ± 0.24 | 10.26 ± 0.35 |
| 22:2n-6 | 0.02 ± 0.01 | 0.03 ± 0 | 0.03 ± 0 |
| 22:4n-6 | 2.79 ± 0.06 | 2.79 ± 0.05 | 2.8 ± 0.03 |
| 22:5n-6 | 0.19 ± 0 | 0.2 ± 0.01 | 0.18 ± 0.01 |
| ***n-6 PUFAs*** | *15.06 ± 0.04* | *15.31 ± 0.03* | *15.3 ± 0.04* |
| 18:3n-3 | 0.01 ± 0.01 | 0.01 ± 0 | 0.01 ± 0 |
| 20:5n-3 | 0.07 ± 0 | 0.07 ± 0 | 0.07 ± 0 |
| 22:5n-3 | 0.19 ± 0 | 0.18 ± 0 | 0.19 ± 0 |
| 22:6n-3 | 18.44 ± 0.2 | 18.38 ± 0.36 | 18.57 ± 0.34 |
| ***n-3 PUFAs*** | *18.71 ± 0.05* | *18.65 ± 0.09* | *18.84 ± 0.08* |

| **Supplemental Table 1D. Brain fatty-acid concentrations of mice fed fish, algal and ^13^C enriched diets at day 5** | | | |
| --- | --- | --- | --- |
|  | Concentration of fatty acids (umol/g) | | |
| Fatty acid | Fish-DHA (n=4) | Algal-DHA (n=4) | ^13^C Enriched-DHA (n=4) |
| 10:0 | 0.01 ± 0 | 0.01 ± 0 | 0.01 ± 0 |
| 12:0 | 0.03 ± 0 | 0.03 ± 0.01 | 0.03 ± 0.05 |
| 14:0 | 0.73 ± 0.21 | 0.58 ± 0.22 | 0.55 ± 0.19 |
| 16:0 | 34.61 ± 1.2 | 32.01 ± 0.75 | 32.22 ± 1.15 |
| 17:0 | 0.23 ± 0.01 | 0.23 ± 0.05 | 0.23 ± 0.01 |
| 18:0 | 26.31 ± 0.78 | 25.48 ± 0.28 | 25.38 ± 0.57 |
| 20:0 | 0.43 ± 0.05 | 0.46 ± 0.03 | 0.48 ± 0.02 |
| 22:0 | 0.27 ± 0.03 | 0.28 ± 0.02 | 0.3 ± 0.01 |
| 23:0 | 0.08 ± 0.01 | 0.08 ± 0 | 0.09 ± 0 |
| 24:0 | 0.28 ± 0.03 | 0.3 ± 0.02 | 0.31 ± 0.01 |
| ***SFAs*** | *62.98 ± 0.13* | *59.46 ± 0.08* | *61.42 ± 0.07* |
| 14:1 | 0.24 ± 0.1 | 0.16 ± 0.1 | 0.15 ± 0.09 |
| 16:1n-7 | 0.98 ± 0.02 | 0.89 ± 0.04 | 0.96 ± 0.05 |
| 16:1n-9 | 0.71 ± 0.21 | 0.52 ± 0.2 | 0.53 ± 0.19 |
| 18:1n-7 | 5.07 ± 0.19 | 4.85 ± 0.11 | 3.79 ± 1.27 |
| 18:1n-9 | 22.51 ± 1.05 | 21.93 ± 0.57 | 22.81 ± 0.78 |
| 20:1n-9 | 2.62 ± 0.25 | 2.8 ± 0.15 | 2.95 ± 0.12 |
| 22:1n-9 | 0.23 ± 0.02 | 0.23 ± 0.01 | 0.26 ± 0.01 |
| 24:1n-9 | 0.3 ± 0.11 | 0.4 ± 0.04 | 0.47 ± 0.04 |
| ***MUFAs*** | *32.65 ± 0.12* | *31.77 ± 0.06* | *32.93 ± 0.16* |
| 18:2n-6 | 0.91 ± 0.02 | 1.03 ± 0.05 | 0.89 ± 0.02 |
| 18:3n-6 | 0.01 ± 0 | 0 ± 0 | 0 ± 0 |
| 20:2n-6 | 0.19 ± 0.01 | 0.32 ± 0 | 0.25 ± 0.02 |
| 20:3n-6 | 0.71 ± 0.02 | 0.73 ± 0.03 | 0.68 ± 0.02 |
| 20:4n-6 | 10.45 ± 0.28 | 10.01 ± 0.07 | 9.9 ± 0.06 |
| 22:2n-6 | 0.02 ± 0 | 0.03 ± 0 | 0.03 ± 0.01 |
| 22:4n-6 | 2.6 ± 0.11 | 2.56 ± 0.07 | 2.67 ± 0.04 |
| 22:5n-6 | 0.17 ± 0.01 | 0.18 ± 0 | 0.18 ± 0.01 |
| ***n-6 PUFAs*** | *15.05 ± 0.03* | *14.86 ± 0.01* | *15.07 ± 0.04* |
| 18:3n-3 | 0.01 ± 0 | 0.02 ± 0.01 | 0.01 ± 0.01 |
| 20:5n-3 | 0.08 ± 0.03 | 0.09 ± 0 | 0.08 ± 0 |
| 22:5n-3 | 0.19 ± 0.01 | 0.19 ± 0 | 0.19 ± 0 |
| 22:6n-3 | 18.73 ± 0.28 | 18.31 ± 0.86 | 17.86 ± 0.4 |
| ***n-3 PUFAs*** | *19.0 ± 0.07* | *18.6 ± 0.02* | *18.7 ± 0.08* |

| **Supplemental Table 1E. Brain fatty-acid concentrations of mice fed fish, algal and ^13^C enriched diets at day 7** | | | |
| --- | --- | --- | --- |
|  | Concentration of fatty acids (umol/g) | | |
| Fatty acid | Fish-DHA (n=4) | Algal-DHA (n=4) | ^13^C Enriched-DHA (n=4) |
| 10:0 | 0.01 ± 0 | 0.01 ± 0 | 0.01 ± 0 |
| 12:0 | 0.03 ± 0.01 | 0.04 ± 0.01 | 0.07 ± 0.02 |
| 14:0 | 0.86 ± 0.03 | 0.79 ± 0.05 | 0.96 ± 0.12 |
| 16:0 | 30.97 ± 0.2 | 31.72 ± 1.14 | 31.28 ± 1.54 |
| 17:0 | 0.22 ± 0.01 | 0.22 ± 0.01 | 0.21 ± 0.01 |
| 18:0 | 25.18 ± 0.53 | 25.31 ± 0.62 | 24.37 ± 0.96 |
| 20:0 | 0.45 ± 0.07 | 0.39 ± 0.01 | 0.39 ± 0.06 |
| 22:0 | 0.29 ± 0.04 | 0.25 ± 0.01 | 0.25 ± 0.04 |
| 23:0 | 0.08 ± 0.01 | 0.08 ± 0 | 0.08 ± 0.01 |
| 24:0 | 0.31 ± 0.05 | 0.27 ± 0.01 | 0.27 ± 0.04 |
| ***SFAs*** | *58.38 ± 0.05* | *59.08 ± 0.12* | *57.88 ± 0.17* |
| 14:1 | 0.3 ± 0.01 | 0.27 ± 0.02 | 0.33 ± 0.05 |
| 16:1n-7 | 0.81 ± 0.02 | 0.86 ± 0.05 | 0.96 ± 0.04 |
| 16:1n-9 | 0.83 ± 0.05 | 0.73 ± 0.04 | 0.88 ± 0.12 |
| 18:1n-7 | 4.66 ± 0.22 | 4.62 ± 0.13 | 4.58 ± 0.29 |
| 18:1n-9 | 21.43 ± 1.29 | 20.78 ± 0.52 | 20.54 ± 1.54 |
| 20:1n-9 | 2.73 ± 0.42 | 2.37 ± 0.07 | 2.38 ± 0.4 |
| 22:1n-9 | 0.23 ± 0.03 | 0.21 ± 0.01 | 0.21 ± 0.03 |
| 24:1n-9 | 0.41 ± 0.07 | 0.39 ± 0.02 | 0.34 ± 0.06 |
| ***MUFAs*** | *31.41 ± 0.15* | *30.24 ± 0.06* | *30.22 ± 0.18* |
| 18:2n-6 | 1.06 ± 0.04 | 1.06 ± 0.05 | 0.96 ± 0.06 |
| 18:3n-6 | 0.01 ± 0 | 0.01 ± 0 | 0.01 ± 0 |
| 20:2n-6 | 0.24 ± 0.05 | 0.15 ± 0.02 | 0.11 ± 0.03 |
| 20:3n-6 | 0.75 ± 0.05 | 0.71 ± 0.01 | 0.66 ± 0.04 |
| 20:4n-6 | 10.12 ± 0.25 | 10.36 ± 0.24 | 10.17 ± 0.23 |
| 22:2n-6 | 0.03 ± 0 | 0.02 ± 0 | 0.02 ± 0 |
| 22:4n-6 | 2.68 ± 0.12 | 2.61 ± 0.06 | 2.62 ± 0.08 |
| 22:5n-6 | 0.18 ± 0.01 | 0.19 ± 0.01 | 0.18 ± 0.01 |
| ***n-6 PUFAs*** | *15.04 ± 0.03* | *15.13 ± 0.03* | *14.71 ± 0.03* |
| 18:3n-3 | 0 ± 0 | 0.01 ± 0 | 0 ± 0 |
| 20:5n-3 | 0.08 ± 0.01 | 0.08 ± 0 | 0.07 ± 0.01 |
| 22:5n-3 | 0.19 ± 0.01 | 0.18 ± 0 | 0.18 ± 0.01 |
| 22:6n-3 | 18.7 ± 0.26 | 19.19 ± 0.43 | 18.44 ± 0.31 |
| ***n-3 PUFAs*** | *18.98 ± 0.06* | *19.46 ± 0.11* | *18.69 ± 0.08* |

| **Supplemental Table 1F. Brain fatty-acid concentrations of mice fed fish, algal and ^13^C enriched diets at day 14** | | | |
| --- | --- | --- | --- |
|  | Concentration of fatty acids (umol/g) | | |
| Fatty acid | Fish-DHA (n=4) | Algal-DHA (n=4) | ^13^C Enriched-DHA (n=4) |
| 10:0 | 0.01 ± 0 | 0.01 ± 0 | 0.01 ± 0 |
| 12:0 | 0.04 ± 0.01 | 0.04 ± 0.01 | 0.03 ± 0 |
| 14:0 | 0.91 ± 0.05 | 0.27 ± 0.02 | 0.89 ± 0.21 |
| 16:0 | 29.49 ± 2.44 | 33.47 ± 0.43 | 32.55 ± 1.43 |
| 17:0 | 0.21 ± 0.02 | 0.24 ± 0.01 | 0.23 ± 0.01 |
| 18:0 | 23.48 ± 1.12 | 26.86 ± 0.23 | 25.73 ± 0.92 |
| 20:0 | 0.38 ± 0.04 | 0.45 ± 0.04 | 0.42 ± 0.03 |
| 22:0 | 0.25 ± 0.03 | 0.3 ± 0.02 | 0.28 ± 0.02 |
| 23:0 | 0.07 ± 0.01 | 0.1 ± 0.01 | 0.09 ± 0.01 |
| 24:0 | 0.27 ± 0.04 | 0.32 ± 0.03 | 0.29 ± 0.02 |
| ***SFAs*** | *55.1 ± 0.25* | *62.06 ± 0.04* | *60.51 ± 0.16* |
| 14:1 | 0.33 ± 0.02 | 0 ± 0 | 0.31 ± 0.1 |
| 16:1n-7 | 0.84 ± 0.05 | 1.06 ± 0.05 | 0.92 ± 0.07 |
| 16:1n-9 | 0.9 ± 0.03 | 0.24 ± 0.01 | 0.85 ± 0.2 |
| 18:1n-7 | 4.46 ± 0.2 | 4.91 ± 0.1 | 4.7 ± 0.18 |
| 18:1n-9 | 19.57 ± 1.02 | 22.86 ± 0.74 | 21.71 ± 0.85 |
| 20:1n-9 | 2.33 ± 0.24 | 2.73 ± 0.22 | 2.58 ± 0.18 |
| 22:1n-9 | 0.19 ± 0.02 | 0.23 ± 0.02 | 0.21 ± 0.01 |
| 24:1n-9 | 0.54 ± 0.11 | 0.54 ± 0.09 | 0.49 ± 0.04 |
| ***MUFAs*** | *29.15 ± 0.12* | *32.56 ± 0.09* | *31.76 ± 0.09* |
| 18:2n-6 | 0.96 ± 0.04 | 1.1 ± 0.08 | 0.95 ± 0.04 |
| 18:3n-6 | 0.01 ± 0 | 0.01 ± 0 | 0.01 ± 0 |
| 20:2n-6 | 0.12 ± 0.02 | 0.14 ± 0.03 | 0.12 ± 0.03 |
| 20:3n-6 | 0.63 ± 0.02 | 0.74 ± 0.03 | 0.71 ± 0.02 |
| 20:4n-6 | 9.8 ± 0.23 | 10.74 ± 0.13 | 10.53 ± 0.27 |
| 22:2n-6 | 0 ± 0 | 0.02 ± 0 | 0.02 ± 0.01 |
| 22:4n-6 | 2.51 ± 0.08 | 2.79 ± 0.08 | 2.76 ± 0.03 |
| 22:5n-6 | 0.17 ± 0 | 0.2 ± 0.02 | 0.2 ± 0.01 |
| ***n-6 PUFAs*** | *14.2 ± 0.03* | *15.73 ± 0.02* | *15.3 ± 0.03* |
| 18:3n-3 | 0 ± 0 | 0.02 ± 0 | 0.01 ± 0 |
| 20:5n-3 | 0.14 ± 0.06 | 0.09 ± 0.01 | 0.08 ± 0 |
| 22:5n-3 | 0.16 ± 0 | 0.19 ± 0 | 0.18 ± 0 |
| 22:6n-3 | 17.91 ± 0.33 | 18.59 ± 0.2 | 18.34 ± 0.59 |
| ***n-3 PUFAs*** | *18.21 ± 0.08* | *18.91 ± 0.05* | *18.61 ± 0.15* |

| **Supplemental Table 1G. Brain fatty-acid concentrations of mice fed fish, algal and ^13^C enriched diets at day 28** | | | |
| --- | --- | --- | --- |
|  | Concentration of fatty acids (umol/g) | | |
| Fatty acid | Fish-DHA (n=4) | Algal-DHA (n=4) | ^13^C Enriched-DHA (n=4) |
| 10:0 | 0.01 ± 0 | 0.01 ± 0 | 0.01 ± 0 |
| 12:0 | 0.03 ± 0 | 0.03 ± 0 | 0.03 ± 0 |
| 14:0 | 0.91 ± 0.23 | 0.48 ± 0.23 | 0.65 ± 0.22 |
| 16:0 | 34.5 ± 1.3 | 33.96 ± 0.67 | 33.44 ± 1.25 |
| 17:0 | 0.4 ± 0.02 | 0.37 ± 0.01 | 0.38 ± 0.02 |
| 18:0 | 26.5 ± 0.49 | 26.86 ± 0.38 | 26.88 ± 0.53 |
| 20:0 | 0.46 ± 0.03 | 0.46 ± 0.07 | 0.39 ± 0.03 |
| 22:0 | 0.29 ± 0.02 | 0.3 ± 0.05 | 0.26 ± 0.02 |
| 23:0 | 0.09 ± 0 | 0.1 ± 0.02 | 0.09 ± 0.01 |
| 24:0 | 0.29 ± 0.02 | 0.31 ± 0.06 | 0.28 ± 0.03 |
| ***SFAs*** | *63.49 ± 0.13* | *62.88 ± 0.07* | *62.39 ± 0.13* |
| 14:1 | 0.31 ± 0.12 | 0.11 ± 0.12 | 0.18 ± 0.12 |
| 16:1n-7 | 0.97 ± 0.04 | 1.04 ± 0.01 | 0.96 ± 0.02 |
| 16:1n-9 | 0.93 ± 0.24 | 0.49 ± 0.25 | 0.6 ± 0.23 |
| 18:1n-7 | 5.13 ± 0.11 | 5.16 ± 0.26 | 4.78 ± 0.07 |
| 18:1n-9 | 23.52 ± 0.59 | 23.52 ± 1.31 | 22.37 ± 0.37 |
| 20:1n-9 | 2.99 ± 0.18 | 3.01 ± 0.47 | 2.62 ± 0.23 |
| 22:1n-9 | 0.25 ± 0.01 | 0.27 ± 0.04 | 0.22 ± 0.02 |
| 24:1n-9 | 0.48 ± 0.05 | 0.51 ± 0.09 | 0.47 ± 0.07 |
| ***MUFAs*** | *34.57 ± 0.07* | *34.1 ± 0.15* | *32.2 ± 0.04* |
| 18:2n-6 | 0.91 ± 0.02 | 0.91 ± 0.02 | 1.11 ± 0.04 |
| 18:3n-6 | 0.01 ± 0 | 0.01 ± 0 | 0.01 ± 0 |
| 20:2n-6 | 0.15 ± 0.03 | 0.17 ± 0.04 | 0.16 ± 0.02 |
| 20:3n-6 | 0.68 ± 0.02 | 0.69 ± 0.03 | 0.77 ± 0.02 |
| 20:4n-6 | 10.48 ± 0.15 | 10.97 ± 0.25 | 10.65 ± 0.33 |
| 22:2n-6 | 0.02 ± 0 | 0.02 ± 0.01 | 0.01 ± 0 |
| 22:4n-6 | 2.78 ± 0.06 | 2.9 ± 0.03 | 2.82 ± 0.06 |
| 22:5n-6 | 0.17 ± 0 | 0.19 ± 0.01 | 0.2 ± 0.01 |
| ***n-6 PUFAs*** | *15.19 ± 0.02* | *15.81 ± 0.03* | *15.73 ± 0.04* |
| 18:3n-3 | 0.01 ± 0.01 | 0.01 ± 0 | 0.02 ± 0.01 |
| 20:5n-3 | 0.08 ± 0.03 | 0.07 ± 0 | 0.09 ± 0 |
| 22:5n-3 | 0.18 ± 0 | 0.18 ± 0.01 | 0.21 ± 0 |
| 22:6n-3 | 18.34 ± 0.3 | 18.55 ± 0.15 | 19.22 ± 0.36 |
| ***n-3 PUFAs*** | *18.61 ± 0.07* | *18.82 ± 0.04* | *19.53 ± 0.09* |

| **Supplemental Table 1H. Brain fatty-acid concentrations of mice fed fish, algal and ^13^C enriched diets at day 56** | | | |
| --- | --- | --- | --- |
|  | Concentration of fatty acids (umol/g) | | |
| Fatty acid | Fish-DHA (n=3) | Algal-DHA (n=4) | ^13^C Enriched-DHA (n=4) |
| 10:0 | 0.01 ± 0 | 0.01 ± 0 | 0.01 ± 0 |
| 12:0 | 0.43 ± 0.41 | 0.03 ± 0.01 | 0.03 ± 0 |
| 14:0 | 1.6 ± 0.36 | 1.13 ± 0.12 | 0.71 ± 0.26 |
| 16:0 | 35.39 ± 1.48 | 34.16 ± 1.15 | 33.01 ± 1.39 |
| 17:0 | 0.48 ± 0.01 | 0.52 ± 0.03 | 0.5 ± 0.02 |
| 18:0 | 26.4 ± 0.36 | 26.93 ± 0.44 | 25.99 ± 0.88 |
| 20:0 | 0.49 ± 0.01 | 0.47 ± 0.07 | 0.35 ± 0.02 |
| 22:0 | 0.29 ± 0.03 | 0.29 ± 0.04 | 0.24 ± 0.01 |
| 23:0 | 0.1 ± 0.01 | 0.1 ± 0.01 | 0.09 ± 0 |
| 24:0 | 0.29 ± 0.03 | 0.29 ± 0.04 | 0.25 ± 0.02 |
| ***SFAs*** | *65.48 ± 0.14* | *63.94 ± 0.11* | *61.17 ± 0.15* |
| 14:1 | 0.33 ± 0.13 | 0.42 ± 0.06 | 0.23 ± 0.12 |
| 16:1n-7 | 1.53 ± 0.68 | 0.94 ± 0.01 | 0.92 ± 0.08 |
| 16:1n-9 | 1 ± 0.34 | 1.13 ± 0.04 | 0.73 ± 0.27 |
| 18:1n-7 | 4.77 ± 0.25 | 5.04 ± 0.14 | 4.64 ± 0.08 |
| 18:1n-9 | 25.52 ± 1.38 | 23.71 ± 0.81 | 21.8 ± 0.46 |
| 20:1n-9 | 3.15 ± 0.29 | 3.22 ± 0.43 | 2.47 ± 0.14 |
| 22:1n-9 | 0.23 ± 0.01 | 0.24 ± 0.03 | 0.2 ± 0.01 |
| 24:1n-9 | 0.53 ± 0.05 | 0.52 ± 0.01 | 0.42 ± 0.03 |
| ***MUFAs*** | *37.06 ± 0.16* | *35.21 ± 0.1* | *31.41 ± 0.05* |
| 18:2n-6 | 3.3 ± 2.19 | 1 ± 0.03 | 1.02 ± 0.06 |
| 18:3n-6 | 0.01 ± 0 | 0.01 ± 0 | 0 ± 0 |
| 20:2n-6 | 0.19 ± 0.04 | 0.2 ± 0.02 | 0.18 ± 0.06 |
| 20:3n-6 | 0.75 ± 0.02 | 0.73 ± 0.01 | 0.72 ± 0.03 |
| 20:4n-6 | 9.91 ± 0.17 | 10.72 ± 0.42 | 10.33 ± 0.32 |
| 22:2n-6 | 0.03 ± 0 | 0.02 ± 0 | 0.02 ± 0 |
| 22:4n-6 | 2.64 ± 0.12 | 2.86 ± 0.04 | 2.66 ± 0.04 |
| 22:5n-6 | 0.17 ± 0.01 | 0.19 ± 0.01 | 0.2 ± 0.01 |
| ***n-6 PUFAs*** | *17 ± 0.27* | *15.73 ± 0.05* | *15.13 ± 0.04* |
| 18:3n-3 | 0 ± 0 | 0 ± 0 | 0.01 ± 0.01 |
| 20:5n-3 | 0.09 ± 0 | 0.07 ± 0 | 0.09 ± 0.01 |
| 22:5n-3 | 0.2 ± 0.01 | 0.2 ± 0 | 0.19 ± 0.01 |
| 22:6n-3 | 17.88 ± 0.6 | 18.84 ± 0.43 | 18.68 ± 0.22 |
| ***n-3 PUFAs*** | *18.17 ± 0.15* | *19.12 ± 0.11* | *18.97 ± 0.05* |

| **Supplemental Table 1I. Brain fatty-acid concentrations of mice fed fish, algal and ^13^C enriched diets at day 112** | | | |
| --- | --- | --- | --- |
|  | Concentration of fatty acids (umol/g) | | |
| Fatty acid | Fish-DHA (n=4) | Algal-DHA (n=4) | 13C Enriched-DHA (n=4) |
| 10:0 | 0 ± 0 | 0.01 ± 0 | 0 ± 0 |
| 12:0 | 0.01 ± 0 | 0.01 ± 0 | 0.01 ± 0 |
| 14:0 | 0.16 ± 0.01 | 0.12 ± 0.02 | 0.21 ± 0.09 |
| 16:0 | 24.9 ± 0.84 | 20.49 ± 2.94 | 16.48 ± 1.92 |
| 17:0 | 0.19 ± 0 | 0.17 ± 0.02 | 0.13 ± 0.02 |
| 18:0 | 23.64 ± 0.23 | 21.03 ± 2.23 | 18.1 ± 1.67 |
| 20:0 | 0.4 ± 0.03 | 0.41 ± 0.04 | 0.4 ± 0 |
| 22:0 | 0.33 ± 0.03 | 0.37 ± 0.02 | 0.4 ± 0.02 |
| 23:0 | 0.12 ± 0.01 | 0.13 ± 0.01 | 0.16 ± 0.02 |
| 24:0 | 0.39 ± 0.03 | 0.44 ± 0.01 | 0.49 ± 0.04 |
| ***SFAs*** | *50.15 ± 0.08* | *43.19 ± 0.35* | *36.37 ± 0.24* |
| 14:1 | 0.01 ± 0 | 0.01 ± 0 | 0.05 ± 0.04 |
| 16:1n-7 | 0.76 ± 0.02 | 0.64 ± 0 | 0.46 ± 0.06 |
| 16:1n-9 | 0.16 ± 0.01 | 0.13 ± 0.02 | 0.3 ± 0.14 |
| 18:1n-7 | 4.25 ± 0.02 | 3.82 ± 0.48 | 3.18 ± 0.28 |
| 18:1n-9 | 20.23 ± 0.19 | 18.12 ± 2.28 | 15.17 ± 1.35 |
| 20:1n-9 | 2.8 ± 0.22 | 2.76 ± 0.31 | 2.58 ± 0.06 |
| 22:1n-9 | 0.23 ± 0.02 | 0.23 ± 0.01 | 0.24 ± 0.01 |
| 24:1n-9 | 0.02 ± 0 | 0.01 ± 0 | 0.03 ± 0.01 |
| ***MUFAs*** | *28.45 ± 0.03* | *25.73 ± 0.28* | *22.03 ± 0.16* |
| 18:2n-6 | 0.9 ± 0.03 | 0.74 ± 0.08 | 0.66 ± 0.08 |
| 18:3n-6 | 0 ± 0 | 0 ± 0 | 0 ± 0 |
| 20:2n-6 | 0.19 ± 0.01 | 0.17 ± 0.02 | 0.17 ± 0.01 |
| 20:3n-6 | 0.69 ± 0.02 | 0.62 ± 0.05 | 0.57 ± 0.04 |
| 20:4n-6 | 9.29 ± 0.1 | 8.81 ± 0.6 | 7.76 ± 0.48 |
| 22:2n-6 | 0.03 ± 0 | 0.03 ± 0 | 0.03 ± 0 |
| 22:4n-6 | 2.6 ± 0.05 | 2.67 ± 0.09 | 2.62 ± 0.04 |
| 22:5n-6 | 0.16 ± 0 | 0.15 ± 0 | 0.15 ± 0 |
| ***n-6 PUFAs*** | *13.86 ± 0.01* | *13.21 ± 0.07* | *11.97 ± 0.06* |
| 18:3n-3 | 0 ± 0 | 0 ± 0 | 0 ± 0 |
| 20:5n-3 | 0.08 ± 0 | 0.06 ± 0 | 0.06 ± 0 |
| 22:5n-3 | 0.2 ± 0 | 0.19 ± 0.01 | 0.18 ± 0 |
| 22:6n-3 | 18.74 ± 0.16 | 18.46 ± 0.35 | 17.75 ± 0.3 |
| ***n-3 PUFAs*** | *19.01 ± 0.04* | *18.71 ± 0.09* | *17.99 ± 0.07* |

| **Supplemental Table 1J. Brain fatty-acid concentrations of mice fed fish, algal and ^13^C enriched diets at day 168** | | | |
| --- | --- | --- | --- |
|  | Concentration of fatty acids (umol/g) | | |
| Fatty acid | Fish-DHA (n=3) | Algal-DHA (n=4) | ^13^C Enriched-DHA (n=4) |
| 10:0 | 0 ± 0 | 0 ± 0 | 0 ± 0 |
| 12:0 | 0.01 ± 0 | 0.01 ± 0 | 0.01 ± 0 |
| 14:0 | 0.11 ± 0.03 | 0.32 ± 0.17 | 0.27 ± 0.19 |
| 16:0 | 19.53 ± 3.83 | 26.74 ± 1.69 | 23.97 ± 2.94 |
| 17:0 | 0.16 ± 0 | 0.2 ± 0.01 | 0.18 ± 0.02 |
| 18:0 | 20.26 ± 2.71 | 24.27 ± 0.56 | 22.95 ± 1.82 |
| 20:0 | 0.37 ± 0.04 | 0.39 ± 0.01 | 0.41 ± 0 |
| 22:0 | 0.35 ± 0.04 | 0.32 ± 0 | 0.36 ± 0.01 |
| 23:0 | 0.14 ± 0.02 | 0.12 ± 0 | 0.14 ± 0 |
| 24:0 | 0.39 ± 0.04 | 0.35 ± 0.01 | 0.41 ± 0.01 |
| ***SFAs*** | *35.27 ± 0.53* | *52.72 ± 0.17* | *48.71 ± 0.32* |
| 14:1 | 0.01 ± 0 | 0.09 ± 0.08 | 0.15 ± 0.08 |
| 16:1n-7 | 0.57 ± 0.12 | 0.8 ± 0.04 | 0.69 ± 0.07 |
| 16:1n-9 | 0.13 ± 0.03 | 0.4 ± 0.22 | 0.6 ± 0.27 |
| 18:1n-7 | 3.7 ± 0.54 | 4.46 ± 0.14 | 4.12 ± 0.35 |
| 18:1n-9 | 17.52 ± 2.51 | 21.22 ± 0.56 | 19.96 ± 1.79 |
| 20:1n-9 | 2.65 ± 0.26 | 2.74 ± 0.05 | 2.84 ± 0.15 |
| 22:1n-9 | 0.39 ± 0.17 | 0.2 ± 0 | 0.22 ± 0 |
| 24:1n-9 | 0.02 ± 0.01 | 0.01 ± 0 | 0 ± 0 |
| ***MUFAs*** | *20.95 ± 0.39* | *29.94 ± 0.07* | *28.58 ± 0.21* |
| 18:2n-6 | 0.63 ± 0.09 | 0.79 ± 0.05 | 0.82 ± 0.07 |
| 18:3n-6 | 0 ± 0 | 0 ± 0 | 0 ± 0 |
| 20:2n-6 | 0.14 ± 0.01 | 0.15 ± 0.01 | 0.17 ± 0.01 |
| 20:3n-6 | 0.59 ± 0.05 | 0.62 ± 0.02 | 0.65 ± 0.04 |
| 20:4n-6 | 8.13 ± 0.7 | 9.94 ± 0.14 | 9.29 ± 0.61 |
| 22:2n-6 | 0.05 ± 0.02 | 0.03 ± 0 | 0.03 ± 0 |
| 22:4n-6 | 2.52 ± 0.04 | 2.69 ± 0.04 | 2.71 ± 0.12 |
| 22:5n-6 | 0.13 ± 0.01 | 0.16 ± 0.01 | 0.16 ± 0.01 |
| ***n-6 PUFAs*** | *11.4 ± 0.12* | *14.39 ± 0.02* | *13.83 ± 0.07* |
| 18:3n-3 | 0 ± 0 | 0 ± 0 | 0 ± 0 |
| 20:5n-3 | 0.06 ± 0 | 0.06 ± 0 | 0.06 ± 0.01 |
| 22:5n-3 | 0.19 ± 0 | 0.18 ± 0.01 | 0.2 ± 0.01 |
| 22:6n-3 | 16.82 ± 0.81 | 18.46 ± 0.2 | 18.52 ± 0.68 |
| ***n-3 PUFAs*** | *17.57 ± 0.08* | *18.7 ± 0.05* | *18.78 ± 0.17* |

| **Supplemental Table 2A. Liver fatty-acid concentrations of mice fed fish-DHA diet (control) at baseline** | |
| --- | --- |
|  | Concentration of fatty acids (umol/g) |
| Fatty acid | Fish-DHA (n=6) |
| 10:0 | 0.01 ± 0 |
| 12:0 | 0.72 ± 0.04 |
| 14:0 | 2.69 ± 0.19 |
| 16:0 | 57.88 ± 3.5 |
| 17:0 | 0.49 ± 0.03 |
| 18:0 | 13.26 ± 0.4 |
| 20:0 | 1.42 ± 0.11 |
| 22:0 | 0.3 ± 0.02 |
| 23:0 | 0.02 ± 0 |
| 24:0 | 0.05 ± 0 |
| ***SFAs*** | *76.85 ± 0.34* |
| 14:1 | 0.11 ± 0.01 |
| 16:1n-7 | 7.06 ± 0.63 |
| 16:1n-9 | 1.07 ± 0.1 |
| 18:1n-7 | 4.26 ± 0.42 |
| 18:1n-9 | 37.06 ± 3.32 |
| 20:1n-9 | 1.15 ± 0.1 |
| 22:1n-9 | 0.15 ± 0.02 |
| ***MUFAs*** | *50.86 ± 0.45* |
| 18:2n-6 | 48.36 ± 3.31 |
| 18:3n-6 | 0.74 ± 0.08 |
| 20:2n-6 | 0.51 ± 0.04 |
| 20:3n-6 | 2.04 ± 0.13 |
| 20:4n-6 | 9.55 ± 0.53 |
| 22:2n-6 | 0.1 ± 0.03 |
| 22:4n-6 | 0.57 ± 0.06 |
| 22:5n-6 | 0.34 ± 0.04 |
| ***n-6 PUFAs*** | *62.21 ± 0.4* |
| 18:3n-3 | 0.04 ± 0 |
| 20:5n-3 | 0.41 ± 0.04 |
| 22:5n-3 | 0.56 ± 0.05 |
| 22:6n-3 | 17.64 ± 0.8 |
| ***n-3 PUFAs*** | *18.66 ± 0.19* |

| **Supplemental Table 2B. Liver fatty-acid concentrations of mice fed fish, algal and spiked diets at day 1** | | | |
| --- | --- | --- | --- |
|  | Concentration of fatty acids (umol/g) | | |
| Fatty acid | Fish-DHA (n=4) | Algal-DHA (n=4) | ^13^C Enriched-DHA (n=4) |
| 10:0 | 0.02 ± 0 | 0.01 ± 0 | 0.03 ± 0 |
| 12:0 | 0.82 ± 0.08 | 0.69 ± 0.08 | 1.3 ± 0.07 |
| 14:0 | 2.71 ± 0.19 | 2.93 ± 0.33 | 3.99 ± 0.26 |
| 16:0 | 62.32 ± 2.66 | 57.75 ± 5.17 | 65.22 ± 5.33 |
| 17:0 | 0.48 ± 0.02 | 0.43 ± 0.03 | 0.44 ± 0.04 |
| 18:0 | 15.11 ± 0.07 | 13.24 ± 1.19 | 14.4 ± 0.66 |
| 20:0 | 1.39 ± 0.13 | 1.36 ± 0.11 | 1.53 ± 0.16 |
| 22:0 | 0.31 ± 0.04 | 0.28 ± 0.02 | 0.32 ± 0.03 |
| 23:0 | 0.04 ± 0 | 0.04 ± 0.01 | 0.04 ± 0.01 |
| 24:0 | 0.07 ± 0.01 | 0.06 ± 0 | 0.07 ± 0 |
| ***SFAs*** | *83.26 ± 0.26* | *76.81 ± 0.51* | *87.35 ± 0.52* |
| 14:1 | 0.12 ± 0.01 | 0.15 ± 0.02 | 0.22 ± 0.02 |
| 16:1n-7 | 7.17 ± 0.51 | 8.43 ± 1.11 | 10.24 ± 0.99 |
| 16:1n-9 | 1.09 ± 0.04 | 1.26 ± 0.13 | 1.45 ± 0.14 |
| 18:1n-7 | 4.81 ± 0.3 | 5.91 ± 0.71 | 6.34 ± 0.59 |
| 18:1n-9 | 39.35 ± 2.24 | 40.51 ± 4.3 | 48.34 ± 4.65 |
| 20:1n-9 | 1.13 ± 0.06 | 1.25 ± 0.13 | 1.56 ± 0.17 |
| 22:1n-9 | 0.15 ± 0.01 | 0.17 ± 0.02 | 0.24 ± 0.02 |
| ***MUFAs*** | *53.82 ± 0.31* | *57.68 ± 0.58* | *68.38 ± 0.63* |
| 18:2n-6 | 47.99 ± 1.81 | 40.16 ± 2.95 | 43.91 ± 4.3 |
| 18:3n-6 | 0.76 ± 0.04 | 0.55 ± 0.05 | 0.55 ± 0.08 |
| 20:2n-6 | 0.52 ± 0.02 | 0.47 ± 0.04 | 0.55 ± 0.06 |
| 20:3n-6 | 2.14 ± 0.04 | 2.3 ± 0.22 | 2.5 ± 0.16 |
| 20:4n-6 | 12.63 ± 0.3 | 9.86 ± 0.88 | 9.75 ± 0.4 |
| 22:2n-6 | 0.34 ± 0.08 | 0.27 ± 0.08 | 0.3 ± 0.06 |
| 22:4n-6 | 0.82 ± 0.02 | 0.49 ± 0.03 | 0.48 ± 0.06 |
| 22:5n-6 | 0.54 ± 0.01 | 0.3 ± 0.02 | 0.29 ± 0.03 |
| ***n-6 PUFAs*** | *65.73 ± 0.22* | *54.41 ± 0.36* | *58.34 ± 0.52* |
| 18:3n-3 | 0.04 ± 0 | 0.04 ± 0 | 0.04 ± 0 |
| 20:5n-3 | 0.43 ± 0.03 | 0.36 ± 0.01 | 0.37 ± 0.04 |
| 22:5n-3 | 0.57 ± 0.04 | 0.44 ± 0.01 | 0.41 ± 0.05 |
| 22:6n-3 | 18.72 ± 0.71 | 15.55 ± 0.98 | 15.76 ± 0.97 |
| ***n-3 PUFAs*** | *19.77 ± 0.17* | *16.38 ± 0.24* | *16.58 ± 0.24* |

| **Supplemental Table 2C. Liver fatty-acid concentrations of mice fed fish, algal and ^13^C enriched diets at day 3** | | | |
| --- | --- | --- | --- |
|  | Concentration of fatty acids (umol/g) | | |
| Fatty acid | Fish-DHA (n=4) | Algal-DHA (n=4) | ^13^C Enriched-DHA (n=4) |
| 10:0 | 0.03 ± 0.01 | 0.02 ± 0 | 0.03 ± 0.01 |
| 12:0 | 1 ± 0.17 | 0.76 ± 0.15 | 0.97 ± 0.2 |
| 14:0 | 2.82 ± 0.18 | 2.34 ± 0.43 | 3.57 ± 0.57 |
| 16:0 | 53.59 ± 3.6 | 50.2 ± 7.17 | 68.56 ± 11.85 |
| 17:0 | 0.41 ± 0.04 | 0.3 ± 0.04 | 0.43 ± 0.04 |
| 18:0 | 13.09 ± 0.77 | 11.79 ± 0.77 | 13.52 ± 1.38 |
| 20:0 | 1.09 ± 0.12 | 0.83 ± 0.14 | 1.42 ± 0.35 |
| 22:0 | 0.23 ± 0.02 | 0.2 ± 0.02 | 0.31 ± 0.07 |
| 23:0 | 0.09 ± 0.02 | 0.06 ± 0.02 | 0.04 ± 0 |
| 24:0 | 0.05 ± 0 | 0.04 ± 0 | 0.06 ± 0.01 |
| ***SFAs*** | *72.39 ± 0.35* | *66.55 ± 0.7* | *88.9 ± 0.9* |
| 14:1 | 0.13 ± 0.01 | 0.12 ± 0.03 | 0.22 ± 0.04 |
| 16:1n-7 | 7.26 ± 0.38 | 6.37 ± 1.19 | 11.5 ± 2.37 |
| 16:1n-9 | 1.02 ± 0.08 | 0.95 ± 0.14 | 1.57 ± 0.26 |
| 18:1n-7 | 4.53 ± 0.21 | 4.12 ± 0.64 | 7.91 ± 1.06 |
| 18:1n-9 | 36.05 ± 3.15 | 33.92 ± 5.82 | 55.06 ± 9.98 |
| 20:1n-9 | 1.02 ± 0.09 | 0.89 ± 0.13 | 1.55 ± 0.26 |
| 22:1n-9 | 0.11 ± 0.01 | 0.1 ± 0.01 | 0.21 ± 0.04 |
| ***MUFAs*** | *50.13 ± 0.43* | *46.47 ± 0.8* | *78.02 ± 1.37* |
| 18:2n-6 | 43.03 ± 5.43 | 36.76 ± 6.06 | 45.33 ± 10.11 |
| 18:3n-6 | 0.63 ± 0.11 | 0.6 ± 0.15 | 0.65 ± 0.18 |
| 20:2n-6 | 0.4 ± 0.05 | 0.36 ± 0.04 | 0.57 ± 0.09 |
| 20:3n-6 | 1.98 ± 0.21 | 1.7 ± 0.12 | 2.14 ± 0.32 |
| 20:4n-6 | 9.89 ± 0.36 | 10.2 ± 0.48 | 10.49 ± 0.5 |
| 22:2n-6 | 1.01 ± 0.26 | 0.68 ± 0.17 | 0.32 ± 0.03 |
| 22:4n-6 | 0.52 ± 0.04 | 0.51 ± 0.09 | 0.61 ± 0.12 |
| 22:5n-6 | 0.33 ± 0.02 | 0.33 ± 0.06 | 0.39 ± 0.04 |
| ***n-6 PUFAs*** | *57.8 ± 0.66* | *51.15 ± 0.74* | *60.49 ± 1.24* |
| 18:3n-3 | 0.04 ± 0 | 0.03 ± 0.01 | 0.03 ± 0.01 |
| 20:5n-3 | 0.33 ± 0.06 | 0.31 ± 0.06 | 0.38 ± 0.15 |
| 22:5n-3 | 0.45 ± 0.08 | 0.39 ± 0.08 | 0.51 ± 0.19 |
| 22:6n-3 | 15.59 ± 1.29 | 14.28 ± 1.47 | 17.01 ± 2.86 |
| ***n-3 PUFAs*** | *16.4 ± 0.31* | *15.01 ± 0.36* | *17.94 ± 0.69* |

| **Supplemental Table 2D. Liver fatty-acid concentrations of mice fed fish, algal and ^13^C enriched diets at day 5** | | | |
| --- | --- | --- | --- |
|  | Concentration of fatty acids (umol/g) | | |
| Fatty acid | Fish-DHA (n=4) | Algal-DHA (n=4) | ^13^C Enriched-DHA (n=4) |
| 10:0 | 0.04 ± 0.01 | 0.03 ± 0.01 | 0.03 ± 0 |
| 12:0 | 1.31 ± 0.32 | 1.04 ± 0.42 | 1.01 ± 0.06 |
| 14:0 | 3.62 ± 0.39 | 2.05 ± 0.58 | 2.81 ± 0.19 |
| 16:0 | 69.49 ± 6.68 | 44.13 ± 6.36 | 57.78 ± 5.02 |
| 17:0 | 0.53 ± 0.03 | 0.35 ± 0.04 | 0.5 ± 0.02 |
| 18:0 | 14.46 ± 0.6 | 13.06 ± 0.76 | 13.8 ± 0.53 |
| 20:0 | 1.62 ± 0.07 | 1.14 ± 0.13 | 1.17 ± 0.19 |
| 22:0 | 0.36 ± 0.01 | 0.35 ± 0.02 | 0.32 ± 0.03 |
| 23:0 | 0.04 ± 0.01 | 0.04 ± 0.01 | 0.03 ± 0 |
| 24:0 | 0.06 ± 0 | 0.08 ± 0.01 | 0.06 ± 0.01 |
| ***SFAs*** | *91.53 ± 0.66* | *62.27 ± 0.62* | *77.5 ± 0.49* |
| 14:1 | 0.12 ± 0.04 | 0.05 ± 0.02 | 0.13 ± 0.01 |
| 16:1n-7 | 9.69 ± 1.22 | 4.38 ± 1.3 | 7.4 ± 0.46 |
| 16:1n-9 | 1.45 ± 0.23 | 0.68 ± 0.24 | 0.94 ± 0.09 |
| 18:1n-7 | 5.81 ± 1.27 | 2.66 ± 0.73 | 3.99 ± 0.34 |
| 18:1n-9 | 50.16 ± 5.39 | 26.54 ± 7.5 | 37.86 ± 3.54 |
| 20:1n-9 | 1.5 ± 0.1 | 0.94 ± 0.25 | 1.1 ± 0.1 |
| 22:1n-9 | 0.2 ± 0.01 | 0.15 ± 0.03 | 0.15 ± 0.02 |
| ***MUFAs*** | *68.93 ± 0.73* | *35.41 ± 1.02* | *51.57 ± 0.49* |
| 18:2n-6 | 57.11 ± 1.93 | 37.86 ± 6.84 | 49.6 ± 3.71 |
| 18:3n-6 | 0.89 ± 0.04 | 0.71 ± 0.14 | 0.82 ± 0.07 |
| 20:2n-6 | 0.61 ± 0.04 | 0.49 ± 0.09 | 0.46 ± 0.04 |
| 20:3n-6 | 2.35 ± 0.15 | 1.66 ± 0.23 | 1.88 ± 0.12 |
| 20:4n-6 | 10.65 ± 0.9 | 10.28 ± 0.78 | 10.59 ± 0.49 |
| 22:2n-6 | 0.5 ± 0.15 | 0.49 ± 0.19 | 0.21 ± 0.06 |
| 22:4n-6 | 0.72 ± 0.1 | 0.62 ± 0.16 | 0.63 ± 0.1 |
| 22:5n-6 | 0.4 ± 0.05 | 0.44 ± 0.07 | 0.43 ± 0.07 |
| ***n-6 PUFAs*** | *73.24 ± 0.24* | *52.54 ± 0.83* | *64.62 ± 0.45* |
| 18:3n-3 | 0.04 ± 0 | 0.02 ± 0 | 0.04 ± 0 |
| 20:5n-3 | 0.57 ± 0.05 | 0.27 ± 0.09 | 0.44 ± 0.02 |
| 22:5n-3 | 0.8 ± 0.05 | 0.44 ± 0.11 | 0.59 ± 0.04 |
| 22:6n-3 | 20.38 ± 0.38 | 17.36 ± 1.29 | 18.29 ± 0.91 |
| ***n-3 PUFAs*** | *21.79 ± 0.09* | *18.1 ± 0.31* | *19.36 ± 0.22* |

| **Supplemental Table 2E. Liver fatty-acid concentrations of mice fed fish, algal and ^13^C enriched diets at day 7** | | | |
| --- | --- | --- | --- |
|  | Concentration of fatty acids (umol/g) | | |
| Fatty acid | Fish-DHA (n=4) | Algal-DHA (n=4) | ^13^C Enriched-DHA (n=4) |
| 10:0 | 0.01 ± 0 | 0.01 ± 0 | 0.02 ± 0.01 |
| 12:0 | 0.72 ± 0.25 | 0.49 ± 0.09 | 1.31 ± 0.22 |
| 14:0 | 1.81 ± 0.54 | 1.51 ± 0.08 | 4.05 ± 0.52 |
| 16:0 | 36.64 ± 7.59 | 35.03 ± 1.79 | 72.25 ± 11.13 |
| 17:0 | 0.31 ± 0.05 | 0.29 ± 0.02 | 0.54 ± 0.07 |
| 18:0 | 12.65 ± 0.88 | 11.69 ± 0.36 | 14.19 ± 0.95 |
| 20:0 | 0.94 ± 0.11 | 1.08 ± 0.16 | 1.8 ± 0.34 |
| 22:0 | 0.29 ± 0 | 0.36 ± 0.03 | 0.41 ± 0.05 |
| 23:0 | 0.02 ± 0 | 0.03 ± 0 | 0.03 ± 0 |
| 24:0 | 0.06 ± 0.01 | 0.08 ± 0.01 | 0.08 ± 0.01 |
| ***SFAs*** | *53.46 ± 0.74* | *50.57 ± 0.17* | *94.68 ± 1.09* |
| 14:1 | 0.02 ± 0 | 0.02 ± 0 | 0.02 ± 0 |
| 16:1n-7 | 3.42 ± 1.15 | 3.05 ± 0.22 | 11.8 ± 1.86 |
| 16:1n-9 | 0.63 ± 0.24 | 0.47 ± 0.07 | 1.53 ± 0.33 |
| 18:1n-7 | 2.18 ± 0.55 | 2.32 ± 0.26 | 6.93 ± 1.08 |
| 18:1n-9 | 21.3 ± 8.12 | 18.16 ± 2.12 | 56.43 ± 10.3 |
| 20:1n-9 | 0.79 ± 0.25 | 0.67 ± 0.11 | 1.71 ± 0.33 |
| 22:1n-9 | 0.17 ± 0.02 | 0.16 ± 0.01 | 0.29 ± 0.05 |
| ***MUFAs*** | *28.5 ± 1.12* | *24.86 ± 0.29* | *78.7 ± 1.41* |
| 18:2n-6 | 33.29 ± 9.73 | 25.44 ± 2.16 | 56.76 ± 9.69 |
| 18:3n-6 | 0.54 ± 0.18 | 0.37 ± 0.04 | 0.89 ± 0.17 |
| 20:2n-6 | 0.42 ± 0.09 | 0.36 ± 0.04 | 0.74 ± 0.08 |
| 20:3n-6 | 1.51 ± 0.24 | 1.53 ± 0.13 | 2.48 ± 0.38 |
| 20:4n-6 | 9.1 ± 1.09 | 9.01 ± 0.4 | 10.3 ± 0.31 |
| 22:2n-6 | 0.06 ± 0.02 | 0.2 ± 0.03 | 0.13 ± 0.04 |
| 22:4n-6 | 0.47 ± 0.13 | 0.46 ± 0.06 | 0.85 ± 0.14 |
| 22:5n-6 | 0.31 ± 0.07 | 0.31 ± 0.04 | 0.44 ± 0.03 |
| ***n-6 PUFAs*** | *45.7 ± 1.19* | *37.68 ± 0.26* | *72.59 ± 1.19* |
| 18:3n-3 | 0.02 ± 0.01 | 0.01 ± 0 | 0.05 ± 0.01 |
| 20:5n-3 | 0.29 ± 0.11 | 0.17 ± 0.02 | 0.56 ± 0.14 |
| 22:5n-3 | 0.39 ± 0.14 | 0.28 ± 0.04 | 0.82 ± 0.21 |
| 22:6n-3 | 15.71 ± 1.97 | 14.55 ± 0.66 | 21.13 ± 2.5 |
| ***n-3 PUFAs*** | *16.41 ± 0.47* | *15.01 ± 0.16* | *22.56 ± 0.59* |

| **Supplemental Table 2F. Liver fatty-acid concentrations of mice fed fish, algal and ^13^C enriched diets at day 14** | | | |
| --- | --- | --- | --- |
|  | Concentration of fatty acids (umol/g) | | |
| Fatty acid | Fish-DHA (n=4) | Algal-DHA (n=4) | ^13^C Enriched-DHA (n=4) |
| 10:0 | 0.01 ± 0 | 0.02 ± 0 | 0.02 ± 0 |
| 12:0 | 0.77 ± 0.04 | 0.8 ± 0.06 | 0.67 ± 0.13 |
| 14:0 | 2.56 ± 0.14 | 2.17 ± 0.24 | 1.81 ± 0.21 |
| 16:0 | 52.3 ± 4.6 | 43.45 ± 3.46 | 39.6 ± 2.7 |
| 17:0 | 0.48 ± 0.05 | 0.33 ± 0.04 | 0.39 ± 0.05 |
| 18:0 | 12.8 ± 0.44 | 11.52 ± 0.24 | 11.05 ± 0.62 |
| 20:0 | 1.32 ± 0.18 | 0.67 ± 0.23 | 1.11 ± 0.07 |
| 22:0 | 0.32 ± 0.03 | 0.22 ± 0.03 | 0.35 ± 0.03 |
| 23:0 | 0.03 ± 0 | 0.04 ± 0.01 | 0.02 ± 0 |
| 24:0 | 0.05 ± 0 | 0.05 ± 0 | 0.07 ± 0 |
| ***SFAs*** | *70.65 ± 0.45* | *59.27 ± 0.34* | *55.08 ± 0.26* |
| 14:1 | 0.02 ± 0 | 0.02 ± 0 | 0.02 ± 0 |
| 16:1n-7 | 6.35 ± 0.51 | 5.38 ± 0.81 | 3.68 ± 0.47 |
| 16:1n-9 | 1.07 ± 0.12 | 0.67 ± 0.16 | 0.62 ± 0.09 |
| 18:1n-7 | 4.28 ± 0.54 | 2.72 ± 0.46 | 2.42 ± 0.25 |
| 18:1n-9 | 36.04 ± 3.39 | 28.21 ± 4.3 | 24.06 ± 3.38 |
| 20:1n-9 | 1.17 ± 0.09 | 0.73 ± 0.15 | 0.91 ± 0.12 |
| 22:1n-9 | 0.2 ± 0.02 | 0.11 ± 0.03 | 0.19 ± 0.01 |
| ***MUFAs*** | *49.13 ± 0.46* | *37.85 ± 0.59* | *31.9 ± 0.46* |
| 18:2n-6 | 47.57 ± 3.59 | 36.07 ± 4.44 | 35.98 ± 5.24 |
| 18:3n-6 | 0.73 ± 0.07 | 0.73 ± 0.13 | 0.59 ± 0.1 |
| 20:2n-6 | 0.56 ± 0.05 | 0.33 ± 0.07 | 0.46 ± 0.04 |
| 20:3n-6 | 2.12 ± 0.11 | 1.46 ± 0.21 | 1.71 ± 0.12 |
| 20:4n-6 | 10.33 ± 0.31 | 10.24 ± 0.32 | 8.69 ± 0.56 |
| 22:2n-6 | 0.31 ± 0.05 | 0.28 ± 0.08 | 0.15 ± 0.02 |
| 22:4n-6 | 0.74 ± 0.1 | 0.47 ± 0.09 | 0.53 ± 0.06 |
| 22:5n-6 | 0.42 ± 0.04 | 0.31 ± 0.05 | 0.37 ± 0.03 |
| ***n-6 PUFAs*** | *62.78 ± 0.44* | *49.88 ± 0.54* | *48.48 ± 0.64* |
| 18:3n-3 | 0.03 ± 0 | 0.03 ± 0.01 | 0.03 ± 0 |
| 20:5n-3 | 0.46 ± 0.06 | 0.39 ± 0.07 | 0.33 ± 0.09 |
| 22:5n-3 | 0.66 ± 0.09 | 0.47 ± 0.08 | 0.49 ± 0.1 |
| 22:6n-3 | 18.53 ± 0.79 | 16.72 ± 0.9 | 16.3 ± 1.28 |
| ***n-3 PUFAs*** | *19.68 ± 0.19* | *17.59 ± 0.21* | *17.14 ± 0.3* |

| **Supplemental Table 2G. Liver fatty-acid concentrations of mice fed fish, algal and ^13^C enriched diets at day 28** | | | |
| --- | --- | --- | --- |
|  | Concentration of fatty acids (umol/g) | | |
| Fatty acid | Fish-DHA (n=4) | Algal-DHA (n=4) | ^13^C Enriched-DHA (n=4) |
| 10:0 | 0.02 ± 0 | 0.03 ± 0 | 0.02 ± 0 |
| 12:0 | 0.7 ± 0.12 | 0.88 ± 0.03 | 0.6 ± 0.04 |
| 14:0 | 2.2 ± 0.43 | 2.99 ± 0.17 | 1.5 ± 0.15 |
| 16:0 | 46.61 ± 8.97 | 58.62 ± 4.94 | 37.56 ± 3.38 |
| 17:0 | 0.44 ± 0.07 | 0.56 ± 0.06 | 0.28 ± 0.02 |
| 18:0 | 11.25 ± 1.61 | 13.16 ± 0.65 | 13.08 ± 0.73 |
| 20:0 | 1.1 ± 0.26 | 1.29 ± 0.15 | 0.84 ± 0.18 |
| 22:0 | 0.26 ± 0.05 | 0.27 ± 0.02 | 0.23 ± 0.04 |
| 23:0 | 0.01 ± 0 | 0.03 ± 0 | 0.02 ± 0 |
| 24:0 | 0.05 ± 0.01 | 0.04 ± 0.01 | 0.05 ± 0.01 |
| ***SFAs*** | *62.64 ± 0.88* | *77.86 ± 0.49* | *54.18 ± 0.33* |
| 14:1 | 0.1 ± 0.02 | 0.14 ± 0.01 | 0.05 ± 0.01 |
| 16:1n-7 | 5.79 ± 1.25 | 8.75 ± 1.02 | 3.08 ± 0.6 |
| 16:1n-9 | 0.91 ± 0.22 | 1.32 ± 0.16 | 0.53 ± 0.06 |
| 18:1n-7 | 3.64 ± 0.77 | 6.07 ± 0.63 | 2.21 ± 0.33 |
| 18:1n-9 | 32.42 ± 7.3 | 47.57 ± 5.41 | 22.2 ± 2.26 |
| 20:1n-9 | 1.02 ± 0.23 | 1.48 ± 0.11 | 0.75 ± 0.12 |
| 22:1n-9 | 0.18 ± 0.04 | 0.23 ± 0.02 | 0.14 ± 0.03 |
| ***MUFAs*** | *44.06 ± 1* | *65.57 ± 0.74* | *28.96 ± 0.31* |
| 18:2n-6 | 40.6 ± 8.57 | 49.77 ± 5.53 | 33.22 ± 3.63 |
| 18:3n-6 | 0.6 ± 0.15 | 0.68 ± 0.12 | 0.57 ± 0.11 |
| 20:2n-6 | 0.45 ± 0.09 | 0.61 ± 0.08 | 0.4 ± 0.07 |
| 20:3n-6 | 1.81 ± 0.35 | 2.33 ± 0.17 | 1.58 ± 0.22 |
| 20:4n-6 | 9.05 ± 1.26 | 10.7 ± 0.33 | 9.41 ± 0.3 |
| 22:2n-6 | 0.05 ± 0.01 | 0.11 ± 0.02 | 0.11 ± 0.02 |
| 22:4n-6 | 0.54 ± 0.11 | 0.66 ± 0.06 | 0.43 ± 0.06 |
| 22:5n-6 | 0.32 ± 0.05 | 0.38 ± 0.01 | 0.33 ± 0.04 |
| ***n-6 PUFAs*** | *53.42 ± 1.04* | *65.23 ± 0.68* | *46.04 ± 0.44* |
| 18:3n-3 | 0.03 ± 0.01 | 0.03 ± 0.01 | 0.02 ± 0 |
| 20:5n-3 | 0.4 ± 0.09 | 0.42 ± 0.11 | 0.33 ± 0.01 |
| 22:5n-3 | 0.53 ± 0.12 | 0.58 ± 0.11 | 0.4 ± 0.05 |
| 22:6n-3 | 15.76 ± 2.58 | 16.64 ± 1.3 | 16.15 ± 1.13 |
| ***n-3 PUFAs*** | *16.72 ± 0.63* | *17.67 ± 0.31* | *16.9 ± 0.28* |

| **Supplemental Table 2H. Liver fatty-acid concentrations of mice fed fish, algal and ^13^C enriched diets at day 56** | | | |
| --- | --- | --- | --- |
|  | Concentration of fatty acids (umol/g) | | |
| Fatty acid | Fish-DHA (n=3) | Algal-DHA (n=4) | ^13^C Enriched-DHA (n=4) |
| 10:0 | 0.02 ± 0.01 | 0.03 ± 0 | 0.02 ± 0 |
| 12:0 | 0.67 ± 0.26 | 1.48 ± 0.11 | 0.42 ± 0.09 |
| 14:0 | 1.66 ± 0.54 | 3.75 ± 0.21 | 1.25 ± 0.15 |
| 16:0 | 32.46 ± 6.56 | 67.1 ± 4.02 | 31.68 ± 1.67 |
| 17:0 | 0.27 ± 0.05 | 0.4 ± 0.01 | 0.32 ± 0.04 |
| 18:0 | 13.99 ± 0.3 | 17.2 ± 1.03 | 12.64 ± 0.29 |
| 20:0 | 0.56 ± 0.19 | 1.42 ± 0.14 | 0.71 ± 0.12 |
| 22:0 | 0.23 ± 0.05 | 0.39 ± 0.02 | 0.25 ± 0.04 |
| 23:0 | 0.03 ± 0.01 | 0.02 ± 0.01 | 0.04 ± 0.02 |
| 24:0 | 0.1 ± 0.02 | 0.09 ± 0.01 | 0.06 ± 0 |
| ***SFAs*** | *49.99 ± 0.64* | *91.88 ± 0.4* | *47.37 ± 0.16* |
| 14:1 | 0.07 ± 0.02 | 0.22 ± 0.01 | 0.06 ± 0.01 |
| 16:1n-7 | 3.41 ± 1.61 | 9.96 ± 0.97 | 2.93 ± 0.33 |
| 16:1n-9 | 0.31 ± 0.1 | 1.06 ± 0.08 | 0.3 ± 0.04 |
| 18:1n-7 | 1.86 ± 0.54 | 4.8 ± 0.45 | 1.98 ± 0.17 |
| 18:1n-9 | 16.47 ± 5.75 | 51.91 ± 4.12 | 13.29 ± 1.47 |
| 20:1n-9 | 0.62 ± 0.24 | 1.5 ± 0.17 | 0.49 ± 0.08 |
| 22:1n-9 | 0.07 ± 0.02 | 0.21 ± 0.03 | 0.07 ± 0.02 |
| ***MUFAs*** | *22.81 ± 0.79* | *69.67 ± 0.56* | *19.12 ± 0.2* |
| 18:2n-6 | 23.39 ± 4.72 | 54.65 ± 4.34 | 21.98 ± 2.66 |
| 18:3n-6 | 0.42 ± 0.08 | 1.4 ± 0.15 | 0.42 ± 0.05 |
| 20:2n-6 | 0.29 ± 0.06 | 0.66 ± 0.09 | 0.38 ± 0.05 |
| 20:3n-6 | 0.99 ± 0.28 | 1.98 ± 0.19 | 1.29 ± 0.07 |
| 20:4n-6 | 7.74 ± 1.29 | 12.16 ± 0.82 | 9.12 ± 0.49 |
| 22:2n-6 | 0.24 ± 0.1 | 0.27 ± 0.11 | 0.44 ± 0.15 |
| 22:4n-6 | 0.22 ± 0.05 | 0.75 ± 0.09 | 0.32 ± 0.05 |
| 22:5n-6 | 0.18 ± 0.04 | 0.58 ± 0.1 | 0.27 ± 0.04 |
| ***n-6 PUFAs*** | *33.48 ± 0.58* | *72.45 ± 0.52* | *34.21 ± 0.32* |
| 18:3n-3 | 0.01 ± 0 | 0.04 ± 0 | 0.01 ± 0 |
| 20:5n-3 | 0.16 ± 0.05 | 0.26 ± 0.04 | 0.13 ± 0.02 |
| 22:5n-3 | 0.17 ± 0.05 | 0.38 ± 0.01 | 0.18 ± 0.04 |
| 22:6n-3 | 13 ± 1.31 | 18.37 ± 0.7 | 12.42 ± 0.94 |
| ***n-3 PUFAs*** | *13.34 ± 0.32* | *19.05 ± 0.17* | *12.73 ± 0.23* |

| **Supplemental Table 2I. Liver fatty-acid concentrations of mice fed fish, algal and ^13^C enriched diets at day 112** | | | |
| --- | --- | --- | --- |
|  | Concentration of fatty acids (umol/g) | | |
| Fatty acid | Fish-DHA (n=4) | Algal-DHA (n=4) | ^13^C Enriched-DHA (n=4) |
| 10:0 | 0 ± 0 | 0.01 ± 0 | 0 ± 0 |
| 12:0 | 0.22 ± 0.06 | 0.43 ± 0.3 | 0.09 ± 0.03 |
| 14:0 | 0.86 ± 0.12 | 1.36 ± 0.79 | 0.44 ± 0.11 |
| 16:0 | 24.6 ± 2.19 | 28.1 ± 9.23 | 17.4 ± 2.8 |
| 17:0 | 0.23 ± 0.03 | 0.24 ± 0.07 | 0.19 ± 0.02 |
| 18:0 | 9.73 ± 0.86 | 9.36 ± 1.13 | 9.41 ± 0.33 |
| 20:0 | 0.76 ± 0.04 | 0.67 ± 0.17 | 0.55 ± 0.04 |
| 22:0 | 0.3 ± 0.02 | 0.22 ± 0.03 | 0.22 ± 0.01 |
| 23:0 | 0.02 ± 0 | 0.03 ± 0 | 0.04 ± 0.01 |
| 24:0 | 0.08 ± 0.01 | 0.06 ± 0.01 | 0.06 ± 0.01 |
| ***SFAs*** | *36.8 ± 0.22* | *40.47 ± 0.9* | *28.39 ± 0.28* |
| 14:1 | 0.03 ± 0 | 0.07 ± 0.05 | 0.01 ± 0.01 |
| 16:1n-7 | 1.94 ± 0.17 | 3.7 ± 1.94 | 1.06 ± 0.33 |
| 16:1n-9 | 0.28 ± 0.03 | 0.48 ± 0.22 | 0.18 ± 0.05 |
| 18:1n-7 | 1.65 ± 0.18 | 2.38 ± 0.62 | 1.15 ± 0.23 |
| 18:1n-9 | 13.87 ± 2.09 | 23.18 ± 10.25 | 8.49 ± 2.17 |
| 20:1n-9 | 0.56 ± 0.07 | 0.83 ± 0.3 | 0.37 ± 0.08 |
| 22:1n-9 | 0.11 ± 0.02 | 0.12 ± 0.04 | 0.08 ± 0.01 |
| ***MUFAs*** | *18.44 ± 0.29* | *30.76 ± 1.41* | *11.34 ± 0.3* |
| 18:2n-6 | 19.57 ± 1.29 | 28.4 ± 11.17 | 15.05 ± 2.64 |
| 18:3n-6 | 0.34 ± 0.03 | 0.56 ± 0.32 | 0.23 ± 0.04 |
| 20:2n-6 | 0.31 ± 0.02 | 0.45 ± 0.11 | 0.29 ± 0.05 |
| 20:3n-6 | 1.01 ± 0.05 | 1.4 ± 0.24 | 0.99 ± 0.09 |
| 20:4n-6 | 7.31 ± 0.11 | 8.84 ± 0.78 | 7.64 ± 0.91 |
| 22:2n-6 | 0.06 ± 0.01 | 0.13 ± 0.02 | 0.25 ± 0.06 |
| 22:4n-6 | 0.39 ± 0.05 | 0.49 ± 0.1 | 0.29 ± 0.06 |
| 22:5n-6 | 0.27 ± 0.02 | 0.31 ± 0.05 | 0.22 ± 0.03 |
| ***n-6 PUFAs*** | *29.27 ± 0.16* | *40.58 ± 1.37* | *24.94 ± 0.33* |
| 18:3n-3 | 0.01 ± 0 | 0.02 ± 0.01 | 0.01 ± 0 |
| 20:5n-3 | 0.15 ± 0.03 | 0.22 ± 0.06 | 0.11 ± 0.04 |
| 22:5n-3 | 0.28 ± 0.06 | 0.35 ± 0.1 | 0.19 ± 0.05 |
| 22:6n-3 | 13.6 ± 0.73 | 14.05 ± 2.11 | 11.03 ± 0.89 |
| ***n-3 PUFAs*** | *14.04 ± 0.18* | *14.65 ± 0.52* | *11.34 ± 0.21* |

| **Supplemental Table 2J. Liver fatty-acid concentrations of mice fed fish, algal and ^13^C enriched diets at day 168** | | | |
| --- | --- | --- | --- |
|  | Concentration of fatty acids (umol/g) | | |
| Fatty acid | Fish-DHA (n=3) | Algal-DHA (n=4) | ^13^C Enriched-DHA (n=4) |
| 10:0 | 0 ± 0 | 0 ± 0 | 0 ± 0 |
| 12:0 | 0.33 ± 0.07 | 0.13 ± 0.03 | 0.15 ± 0.05 |
| 14:0 | 1.38 ± 0.2 | 0.78 ± 0.2 | 0.67 ± 0.19 |
| 16:0 | 32.63 ± 4.71 | 22.63 ± 4.29 | 21.29 ± 5.1 |
| 17:0 | 0.3 ± 0.05 | 0.2 ± 0.04 | 0.25 ± 0.07 |
| 18:0 | 9.81 ± 1 | 9.09 ± 0.91 | 9.33 ± 1.84 |
| 20:0 | 0.81 ± 0.1 | 0.57 ± 0.17 | 0.59 ± 0.07 |
| 22:0 | 0.23 ± 0.01 | 0.19 ± 0.04 | 0.24 ± 0.02 |
| 23:0 | 0.03 ± 0 | 0.03 ± 0 | 0.04 ± 0.01 |
| 24:0 | 0.05 ± 0.01 | 0.06 ± 0.01 | 0.07 ± 0.01 |
| ***SFAs*** | *45.57 ± 0.47* | *33.68 ± 0.42* | *32.64 ± 0.52* |
| 14:1 | 0.06 ± 0.01 | 0.04 ± 0.01 | 0.03 ± 0.01 |
| 16:1n-7 | 4.23 ± 0.52 | 2.81 ± 0.73 | 2.01 ± 0.51 |
| 16:1n-9 | 0.58 ± 0.07 | 0.45 ± 0.12 | 0.23 ± 0.05 |
| 18:1n-7 | 3.14 ± 0.12 | 2.88 ± 0.44 | 1.53 ± 0.22 |
| 18:1n-9 | 26.46 ± 2.94 | 18.04 ± 4.26 | 12.76 ± 2.62 |
| 20:1n-9 | 0.92 ± 0.04 | 0.69 ± 0.14 | 0.48 ± 0.07 |
| 22:1n-9 | 0.12 ± 0.01 | 0.1 ± 0.02 | 0.09 ± 0.01 |
| ***MUFAs*** | *35.52 ± 0.41* | *25.01 ± 0.58* | *17.13 ± 0.36* |
| 18:2n-6 | 30.42 ± 5.97 | 19.37 ± 4.21 | 18.72 ± 3.93 |
| 18:3n-6 | 0.42 ± 0.12 | 0.27 ± 0.08 | 0.32 ± 0.1 |
| 20:2n-6 | 0.5 ± 0.04 | 0.37 ± 0.04 | 0.29 ± 0.03 |
| 20:3n-6 | 1.68 ± 0.12 | 1.36 ± 0.14 | 1.02 ± 0.08 |
| 20:4n-6 | 8.16 ± 0.7 | 9.33 ± 0.66 | 7.64 ± 0.78 |
| 22:2n-6 | 0.15 ± 0.04 | 0.11 ± 0.03 | 0.17 ± 0.08 |
| 22:4n-6 | 0.41 ± 0.01 | 0.42 ± 0.08 | 0.3 ± 0.01 |
| 22:5n-6 | 0.24 ± 0.01 | 0.29 ± 0.03 | 0.23 ± 0.01 |
| ***n-6 PUFAs*** | *41.98 ± 0.73* | *31.52 ± 0.51* | *28.67 ± 0.48* |
| 18:3n-3 | 0.02 ± 0.01 | 0.01 ± 0 | 0.01 ± 0 |
| 20:5n-3 | 0.47 ± 0.14 | 0.19 ± 0.05 | 0.16 ± 0.01 |
| 22:5n-3 | 0.53 ± 0.14 | 0.24 ± 0.07 | 0.24 ± 0.01 |
| 22:6n-3 | 15.71 ± 1.94 | 11.54 ± 1.42 | 12.68 ± 0.32 |
| ***n-3 PUFAs*** | *16.74 ± 0.46* | *11.98 ± 0.35* | *13.09 ± 0.08* |

| **Supplemental Table 3A. Plasma fatty-acid concentrations of mice fed fish-DHA diet (control) at baseline** | |
| --- | --- |
|  | Concentration of fatty acids (nmol/ml) |
| Fatty acid | Fish-DHA (n=6) |
| 10:0 | 18.5 ± 5.9 |
| 12:0 | 370.4 ± 104.9 |
| 14:0 | 266.8 ± 59.9 |
| 16:0 | 2717.6 ± 360.3 |
| 18:0 | 1341.9 ± 173.6 |
| 20:0 | 38 ± 5.7 |
| 22:0 | 14.8 ± 0.9 |
| 24:0 | 3.7 ± 0.4 |
| ***SFAs*** | *4771.7 ± 0* |
| 14:1 | 10 ± 2.4 |
| 16:1n-7 | 258 ± 41.7 |
| 16:1n-9 | 30.3 ± 4.5 |
| 18:1n-7 | 145.9 ± 21.7 |
| 18:1n-9 | 1049.4 ± 143.1 |
| 20:1n-9 | 66.7 ± 12.6 |
| 22:1n-9 | 5.2 ± 0.6 |
| 24:1n-9 | 3.8 ± 0.7 |
| ***MUFAs*** | *1569.3 ± 17.1* |
| 18:2n-6 | 3869.4 ± 549 |
| 18:3n-6 | 46.8 ± 7.5 |
| 20:2n-6 | 18.4 ± 2.2 |
| 20:3n-6 | 129.1 ± 17.4 |
| 20:4n-6 | 1038.4 ± 159.5 |
| 22:4n-6 | 8.9 ± 1.3 |
| 22:5n-6 | 10.2 ± 1.6 |
| ***n-6 PUFAs*** | *5121.3 ± 0* |
| 18:3n-3 | 2.3 ± 0.3 |
| 20:5n-3 | 18.9 ± 1.2 |
| 22:5n-3 | 9 ± 0.9 |
| 22:6n-3 | 791.7 ± 67.2 |
| ***n-3 PUFAs*** | *822 ± 16.6* |

| **Supplemental Table 3B. Plasma fatty-acid concentrations of mice fed fish, algal and C^13^ enriched DHA diets at day 1** | | | |
| --- | --- | --- | --- |
|  | Concentration of fatty acids (nmol/ml) | | |
| Fatty acid | Fish-DHA (n=4) | Algal-DHA (n=4) | C^13^ Enriched-DHA (n=4) |
| 10:0 | 5.3 ± 1.8 | 7.6 ± 1.3 | 10.2 ± 2.6 |
| 12:0 | 218.9 ± 68.8 | 272.9 ± 36 | 400.4 ± 129 |
| 14:0 | 189.5 ± 44.9 | 235.8 ± 26.8 | 338.3 ± 85.6 |
| 16:0 | 2569.3 ± 345.1 | 2485 ± 205.2 | 3240.1 ± 272.7 |
| 18:0 | 1295.8 ± 125 | 1095.5 ± 58.7 | 1441.8 ± 115.9 |
| 20:0 | 29.4 ± 3.7 | 31.2 ± 2.4 | 48 ± 5.6 |
| 22:0 | 11 ± 0.6 | 11.1 ± 1.2 | 17.1 ± 0.4 |
| 24:0 | 2.3 ± 0.3 | 2.4 ± 0.4 | 4.1 ± 0.2 |
| ***SFAs*** | *4321.5 ± 41.8* | *4141.5 ± 0* | *5500.1 ± 0* |
| 14:1 | 7.5 ± 0 | 6.9 ± 1.5 | 8.8 ± 0.8 |
| 16:1n-7 | 208.6 ± 0 | 323.3 ± 36 | 414.2 ± 26.6 |
| 16:1n-9 | 28.7 ± 0 | 38.5 ± 3.8 | 49.2 ± 5.8 |
| 18:1n-7 | 130.6 ± 0 | 208.9 ± 19.5 | 256.2 ± 31.1 |
| 18:1n-9 | 892.5 ± 0 | 1191.8 ± 97.8 | 1641.3 ± 213.3 |
| 20:1n-9 | 58.2 ± 0 | 63.6 ± 9.3 | 94.5 ± 13.6 |
| 22:1n-9 | 3.4 ± 0 | 4.9 ± 0.5 | 7.3 ± 1 |
| 24:1n-9 | 1.6 ± 0 | 1.9 ± 0.5 | 5.2 ± 1.5 |
| ***MUFAs*** | *1331.1 ± 19.3* | *1839.8 ± 0* | *2476.7 ± 0* |
| 18:2n-6 | 3283.9 ± 419 | 3159 ± 313.8 | 4314.7 ± 292.1 |
| 18:3n-6 | 43.9 ± 6.1 | 37.7 ± 2.4 | 46.2 ± 1.1 |
| 20:2n-6 | 17.7 ± 1.6 | 20.5 ± 2.5 | 30.9 ± 4.6 |
| 20:3n-6 | 118.4 ± 15.1 | 142.1 ± 11.6 | 188.5 ± 7 |
| 20:4n-6 | 1130.1 ± 175.1 | 874.4 ± 87.4 | 1069 ± 43.1 |
| 22:4n-6 | 9.7 ± 1.8 | 7.3 ± 1 | 9.9 ± 1.1 |
| 22:5n-6 | 10.9 ± 2.7 | 7.7 ± 1.5 | 11.5 ± 0.8 |
| ***n-6 PUFAs*** | *4614.5 ± 60* | *4248.6 ± 0* | *5670.8 ± 0* |
| 18:3n-3 | 1.5 ± 0 | 1.6 ± 0.1 | 2.1 ± 0.5 |
| 20:5n-3 | 20.1 ± 0 | 22.1 ± 2.8 | 30.2 ± 5.4 |
| 22:5n-3 | 8.5 ± 0 | 7 ± 0.8 | 8.8 ± 0.6 |
| 22:6n-3 | 775.3 ± 0 | 704.1 ± 67.5 | 934.1 ± 24.9 |
| ***n-3 PUFAs*** | *805.4 ± 0* | *734.8 ± 0* | *975.3 ± 0* |

| **Supplemental Table 3C. Plasma fatty-acid concentrations of mice fed fish, algal and C^13^ enriched DHA diets at day 3** | | | |
| --- | --- | --- | --- |
|  | Concentration of fatty acids (nmol/ml) | | |
| Fatty acid | Fish-DHA (n=4) | Algal-DHA (n=4) | C^13^ Enriched-DHA (n=4) |
| 10:0 | 8.5 ± 2.1 | 16.6 ± 9.3 | 6.5 ± 2.3 |
| 12:0 | 385.5 ± 50.3 | 476.6 ± 174 | 206 ± 39 |
| 14:0 | 289.4 ± 31.6 | 310.9 ± 88.8 | 177.4 ± 25.4 |
| 16:0 | 3381.7 ± 264.1 | 3553.8 ± 532.6 | 3079.1 ± 384.7 |
| 18:0 | 1585.7 ± 150 | 1593 ± 194.2 | 1482 ± 142.8 |
| 20:0 | 38.2 ± 2.8 | 33.4 ± 5.3 | 25 ± 2.6 |
| 22:0 | 13.1 ± 1.1 | 15 ± 0.8 | 16.5 ± 1.7 |
| 24:0 | 2.6 ± 0.2 | 3.4 ± 0.2 | 3.8 ± 0.4 |
| ***SFAs*** | *5704.5 ± 0* | *6002.7 ± 0* | *4996.3 ± 0* |
| 14:1 | 4.8 ± 1.2 | 6.3 ± 3.2 | 5.6 ± 2.1 |
| 16:1n-7 | 279.5 ± 28.8 | 258.5 ± 62.5 | 229.2 ± 46.4 |
| 16:1n-9 | 33.5 ± 3.4 | 36.8 ± 8.4 | 26 ± 3.8 |
| 18:1n-7 | 175.1 ± 22.2 | 179.3 ± 42.8 | 175.8 ± 28.3 |
| 18:1n-9 | 1148.1 ± 107.7 | 1234.3 ± 269.9 | 930.4 ± 132.8 |
| 20:1n-9 | 51.8 ± 4.3 | 51.2 ± 10.7 | 37.9 ± 5 |
| 22:1n-9 | 4.3 ± 0.4 | 4.2 ± 0.7 | 2.6 ± 0.4 |
| 24:1n-9 | 2.8 ± 0.8 | 1.9 ± 0 | 1.8 ± 0.3 |
| ***MUFAs*** | *1699.9 ± 0* | *1772.5 ± 0* | *1409.4 ± 0* |
| 18:2n-6 | 4128.2 ± 352.7 | 3919.2 ± 426 | 2910.9 ± 222.8 |
| 18:3n-6 | 47.5 ± 3.9 | 48.7 ± 6.5 | 37.3 ± 3.4 |
| 20:2n-6 | 22.8 ± 1.7 | 28 ± 4.4 | 19.9 ± 1.8 |
| 20:3n-6 | 142.7 ± 8.4 | 151.4 ± 23.7 | 140.8 ± 19.9 |
| 20:4n-6 | 1055.2 ± 99.2 | 1283.1 ± 171 | 920.7 ± 105.1 |
| 22:4n-6 | 10.3 ± 1.5 | 11.6 ± 1.8 | 6.2 ± 1.2 |
| 22:5n-6 | 10.9 ± 2 | 12.9 ± 2.4 | 10 ± 2 |
| ***n-6 PUFAs*** | *5417.7 ± 0* | *5454.8 ± 0* | *4045.8 ± 0* |
| 18:3n-3 | 1.8 ± 0.2 | 2 ± 0.4 | 0.9 ± 0.1 |
| 20:5n-3 | 16.7 ± 2.1 | 19.8 ± 3.9 | 10.9 ± 1.3 |
| 22:5n-3 | 9.1 ± 0.8 | 9.5 ± 1.4 | 6.4 ± 0.8 |
| 22:6n-3 | 884.3 ± 60 | 868 ± 94.8 | 769.7 ± 96.3 |
| ***n-3 PUFAs*** | *911.9 ± 0* | *899.2 ± 0* | *787.9 ± 0* |

| **Supplemental Table 3D. Plasma fatty-acid concentrations of mice fed fish, algal and C^13^ enriched DHA diets at day 5** | | | |
| --- | --- | --- | --- |
|  | Concentration of fatty acids (nmol/ml) | | |
| Fatty acid | Fish-DHA (n=4) | Algal-DHA (n=4) | C^13^ Enriched-DHA (n=4) |
| 10:0 | 17.4 ± 4.7 | 11.7 ± 3.4 | 12.8 ± 6.3 |
| 12:0 | 482.9 ± 90.6 | 372.3 ± 105.4 | 283 ± 84 |
| 14:0 | 358.2 ± 47.7 | 290.6 ± 60.5 | 227.6 ± 19.4 |
| 16:0 | 3733.5 ± 126 | 3198 ± 160.4 | 2902.3 ± 348.6 |
| 18:0 | 1564.6 ± 89.4 | 1462.2 ± 102 | 1284.8 ± 81.6 |
| 20:0 | 43.8 ± 2.2 | 47.4 ± 8.4 | 34.4 ± 0.8 |
| 22:0 | 16.2 ± 0.3 | 16.2 ± 2.1 | 13.2 ± 1.1 |
| 24:0 | 4.4 ± 0.7 | 4.1 ± 0.6 | 3.3 ± 0.7 |
| ***SFAs*** | *6221 ± 0* | *5402.5 ± 0* | *4761.6 ± 0* |
| 14:1 | 8.2 ± 0.8 | 7.3 ± 1.8 | 9 ± 2.8 |
| 16:1n-7 | 398.4 ± 40.8 | 233.6 ± 20.2 | 271 ± 56.6 |
| 16:1n-9 | 48 ± 8.3 | 33.2 ± 4.3 | 29.1 ± 4.4 |
| 18:1n-7 | 224.4 ± 47.7 | 129.7 ± 16.5 | 131.7 ± 18.3 |
| 18:1n-9 | 1594.4 ± 188.1 | 1125.7 ± 71.1 | 1098.2 ± 155 |
| 20:1n-9 | 89.1 ± 1.7 | 59 ± 6.4 | 60.2 ± 6.8 |
| 22:1n-9 | 8.2 ± 0.6 | 6.9 ± 1.1 | 5.9 ± 0.3 |
| C 24:1n-9 | 2.5 ± 1.4 | 1.8 ± 0.7 | 1.8 ± 0.2 |
| ***MUFAs*** | *2373.3 ± 0* | *1597.2 ± 0* | *1606.9 ± 0* |
| 18:2n-6 | 4896.9 ± 65.6 | 4306.6 ± 292.3 | 4082.8 ± 292.7 |
| 18:3n-6 | 49 ± 10.1 | 49.5 ± 12.1 | 45.3 ± 10 |
| 20:2n-6 | 21.4 ± 2 | 21.4 ± 2 | 19.2 ± 3.5 |
| 20:3n-6 | 155.6 ± 17.6 | 118.4 ± 20.7 | 110.5 ± 10.4 |
| 20:4n-6 | 1297.9 ± 146.8 | 1155.7 ± 249.7 | 1103.7 ± 124.2 |
| 22:4n-6 | 12.7 ± 3.3 | 14.3 ± 1.7 | 9.3 ± 1 |
| 22:5n-6 | 14.9 ± 3.4 | 15.7 ± 2.8 | 10.3 ± 1.8 |
| ***n-6 PUFAs*** | *6448.3 ± 0* | *5681.6 ± 0* | *5381.1 ± 0* |
| 18:3n-3 | 2.6 ± 0.3 | 2.6 ± 0.7 | 2.4 ± 0.4 |
| 20:5n-3 | 27.7 ± 2.9 | 17 ± 7.1 | 20.5 ± 3.3 |
| 22:5n-3 | 13.7 ± 1.5 | 9.1 ± 1.4 | 9.8 ± 1.4 |
| 22:6n-3 | 1025 ± 90.4 | 920 ± 78.2 | 843.5 ± 68.7 |
| ***n-3 PUFAs*** | *1069 ± 0* | *948.7 ± 0* | *876.2 ± 0* |

| **Supplemental Table 3E. Plasma fatty-acid concentrations of mice fed fish, algal and C^13^ enriched DHA diets at day 7** | | | |
| --- | --- | --- | --- |
|  | Concentration of fatty acids (nmol/ml) | | |
| Fatty acid | Fish-DHA (n=4) | Algal-DHA (n=4) | C^13^ Enriched-DHA (n=4) |
| 10:0 | 9.8 ± 4.8 | 6.3 ± 3.4 | 11.1 ± 5.2 |
| 12:0 | 245.2 ± 71.1 | 296.7 ± 67.6 | 319.2 ± 108.3 |
| 14:0 | 206.4 ± 34.2 | 285.8 ± 56.2 | 285.1 ± 57.4 |
| 16:0 | 2677.8 ± 121.5 | 2976.7 ± 220.6 | 3325 ± 156.1 |
| 18:0 | 1463.6 ± 59.6 | 1452.9 ± 138.2 | 1474.7 ± 76.8 |
| 20:0 | 43.6 ± 6.6 | 51.9 ± 5.3 | 39.4 ± 2.9 |
| 22:0 | 15.9 ± 2.4 | 17.4 ± 1.7 | 15.2 ± 0.6 |
| 24:0 | 4.3 ± 0.1 | 4.1 ± 0.2 | 3.2 ± 0.3 |
| ***SFAs*** | *4666.6 ± 0* | *5091.9 ± 0* | *5472.7 ± 0* |
| 14:1 | 5.9 ± 1.8 | 6.8 ± 1.6 | 7.9 ± 0.8 |
| 16:1n-7 | 200.9 ± 18.9 | 261.5 ± 41.2 | 406.2 ± 27.5 |
| 16:1n-9 | 30.1 ± 1.1 | 33.4 ± 3.6 | 37.6 ± 1.7 |
| 18:1n-7 | 110.4 ± 7.1 | 165.6 ± 29.5 | 200.1 ± 16.3 |
| 18:1n-9 | 949 ± 58.1 | 1247.6 ± 123 | 1417.1 ± 62.1 |
| 20:1n-9 | 51.1 ± 5.6 | 67 ± 12.8 | 65 ± 10.7 |
| 22:1n-9 | 5.7 ± 0.4 | 6.5 ± 0.9 | 4.9 ± 0.4 |
| 24:1n-9 | 3 ± 0.3 | 1.4 ± 0.6 | 0.7 ± 0.1 |
| ***MUFAs*** | *1356.1 ± 0* | *1789.8 ± 0* | *2139.6 ± 0* |
| 18:2n-6 | 4036 ± 167.6 | 4122.4 ± 292.9 | 4544.7 ± 232.4 |
| 18:3n-6 | 45.4 ± 8.9 | 43.7 ± 9.6 | 56.2 ± 1.8 |
| 20:2n-6 | 16.6 ± 1.6 | 21.4 ± 2.8 | 24.5 ± 3 |
| 20:3n-6 | 116 ± 6.4 | 142.8 ± 9.7 | 159.3 ± 8.3 |
| 20:4n-6 | 1058.5 ± 94.8 | 1091 ± 104.1 | 1260.6 ± 35.9 |
| 22:4n-6 | 11.4 ± 1.7 | 14.7 ± 1.1 | 11.2 ± 1.2 |
| 22:5n-6 | 15.3 ± 3 | 15.6 ± 0.7 | 13.3 ± 2.3 |
| ***n-6 PUFAs*** | *5299.2 ± 0* | *5451.7 ± 0* | *6069.8 ± 0* |
| 18:3n-3 | 2.1 ± 0.7 | 2.7 ± 0.4 | 2.5 ± 0.3 |
| 20:5n-3 | 23 ± 3.1 | 18 ± 1.6 | 26.6 ± 3.2 |
| 22:5n-3 | 8.7 ± 1.6 | 9.2 ± 1.5 | 11.9 ± 0.9 |
| 22:6n-3 | 909.3 ± 52.3 | 866.3 ± 12.3 | 999 ± 29.8 |
| ***n-3 PUFAs*** | *943.1 ± 0* | *896.1 ± 0* | *1040 ± 0* |

| **Supplemental Table 3F. Plasma fatty-acid concentrations of mice fed fish, algal and C^13^ enriched DHA diets at day 14** | | | |
| --- | --- | --- | --- |
|  | Concentration of fatty acids (nmol/ml) | | |
| Fatty acid | Fish-DHA (n=4) | Algal-DHA (n=4) | C^13^ Enriched-DHA (n=4) |
| 10:0 | 1 ± 0.3 | 0.9 ± 0.1 | 1.1 ± 0.5 |
| 12:0 | 44 ± 12.2 | 35.5 ± 26.5 | 72.1 ± 45.5 |
| 14:0 | 105.1 ± 14.9 | 110.1 ± 45.6 | 143.5 ± 57.4 |
| 16:0 | 2696.9 ± 268.1 | 2622.9 ± 91.4 | 2185.1 ± 692.5 |
| 18:0 | 1507 ± 166 | 1217 ± 174.4 | 1185.6 ± 375 |
| 20:0 | 31.5 ± 3.3 | 21.5 ± 4.4 | 30 ± 10 |
| 22:0 | 17.7 ± 1.2 | 16.9 ± 1.3 | 15.5 ± 5.6 |
| 24:0 | 4 ± 0.3 | 5.1 ± 0.3 | 4 ± 1.3 |
| ***SFAs*** | *4407.2 ± 0* | *4029.9 ± 0* | *3636.9 ± 0* |
| 14:1 | 4.1 ± 1.3 | 6.3 ± 1 | 4.3 ± 2.3 |
| 16:1n-7 | 166.4 ± 13.1 | 166.1 ± 20.8 | 110.4 ± 35.6 |
| 16:1n-9 | 21.7 ± 2.4 | 18.4 ± 1 | 16.2 ± 5.3 |
| 18:1n-7 | 117.1 ± 15.1 | 94.8 ± 3.7 | 82.1 ± 22.8 |
| 18:1n-9 | 746.1 ± 79.9 | 877.8 ± 55 | 616.8 ± 181.9 |
| 20:1n-9 | 35.4 ± 1.5 | 39.2 ± 1.5 | 40.2 ± 12.6 |
| 22:1n-9 | 10.6 ± 5.2 | 10 ± 5.4 | 6.4 ± 1.5 |
| C 24:1n-9 | 0.8 ± 0.2 | 0.6 ± 0.1 | 2.2 ± 1.4 |
| ***MUFAs*** | *1102.5 ± 0* | *1213.1 ± 0* | *878.5 ± 0* |
| 18:2n-6 | 3171 ± 332.3 | 2607.8 ± 352.3 | 2712.6 ± 823.4 |
| 18:3n-6 | 41.7 ± 2.5 | 53.4 ± 4.7 | 37.4 ± 11.5 |
| 20:2n-6 | 23.3 ± 1.2 | 20.8 ± 1.1 | 21.6 ± 5.9 |
| 20:3n-6 | 138.8 ± 16 | 103.6 ± 1.2 | 99.4 ± 34.2 |
| 20:4n-6 | 961 ± 80 | 897.4 ± 53.4 | 665.9 ± 208.9 |
| 22:4n-6 | 7.2 ± 1.1 | 7.2 ± 1.1 | 7.1 ± 2 |
| 22:5n-6 | 11.6 ± 0.6 | 10.8 ± 1.6 | 8.5 ± 2 |
| ***n-6 PUFAs*** | *4354.6 ± 0* | *3701.1 ± 0* | *3552.3 ± 0* |
| 18:3n-3 | 0.8 ± 0.3 | 1.3 ± 0.2 | 1.4 ± 0.6 |
| 20:5n-3 | 18.4 ± 4.3 | 22.2 ± 4.7 | 13.7 ± 6.7 |
| 22:5n-3 | 8.2 ± 1.5 | 8.9 ± 1 | 7.2 ± 2.9 |
| 22:6n-3 | 827.7 ± 84.1 | 760.8 ± 12.4 | 670.9 ± 230 |
| ***n-3 PUFAs*** | *855.1 ± 0* | *793.1 ± 0* | *693.3 ± 0* |

| **Supplemental Table 3G. Plasma fatty-acid concentrations of mice fed fish, algal and C^13^ enriched DHA diets at day 28** | | | |
| --- | --- | --- | --- |
|  | Concentration of fatty acids (nmol/ml) | | |
| Fatty acid | Fish-DHA (n=4) | Algal-DHA (n=4) | C^13^ Enriched-DHA (n=4) |
| 10:0 | 1.1 ± 0.1 | 2.5 ± 0.8 | 0.8 ± 0.1 |
| 12:0 | 42.7 ± 25.7 | 132.6 ± 45.4 | 87.4 ± 61.9 |
| 14:0 | 135.6 ± 44.5 | 146.5 ± 30.7 | 106.1 ± 42.5 |
| 16:0 | 2562.6 ± 510 | 2428.2 ± 174.3 | 1916 ± 88 |
| 18:0 | 1380 ± 262.4 | 1275.4 ± 112.9 | 1261.5 ± 101.9 |
| 20:0 | 32.7 ± 6.5 | 29.6 ± 2.4 | 26.2 ± 1.4 |
| 22:0 | 18.6 ± 1.8 | 14.4 ± 1.5 | 13.7 ± 1.4 |
| 24:0 | 5.2 ± 0.7 | 4 ± 0.8 | 4.4 ± 0.4 |
| ***SFAs*** | *4178.5 ± 0* | *4033.2 ± 0* | *3416.1 ± 0* |
| 14:1 | 4.9 ± 0.7 | 4.2 ± 1.7 | 3.1 ± 1.6 |
| 16:1n-7 | 149 ± 32.5 | 187.8 ± 45.8 | 69.3 ± 8.2 |
| 16:1n-9 | 22.7 ± 6.8 | 23.7 ± 4 | 12.5 ± 1.9 |
| 18:1n-7 | 125.8 ± 34.2 | 158.1 ± 20.7 | 67.8 ± 4.2 |
| 18:1n-9 | 785.1 ± 190.1 | 822.1 ± 119.3 | 550.7 ± 56.6 |
| 20:1n-9 | 42.9 ± 5.9 | 47.6 ± 4.5 | 30 ± 4.3 |
| 22:1n-9 | 4.9 ± 0.6 | 6 ± 1.1 | 4.8 ± 0.9 |
| C 24:1n-9 | 1 ± 0.2 | 1.3 ± 0.2 | 1 ± 0.2 |
| ***MUFAs*** | *1136.4 ± 0* | *1250.7 ± 0* | *739.2 ± 0* |
| 18:2n-6 | 2945 ± 619.3 | 2744.8 ± 198.6 | 2479.5 ± 138.9 |
| 18:3n-6 | 38.9 ± 7.3 | 37.1 ± 2.7 | 25.9 ± 4 |
| 20:2n-6 | 25.3 ± 7 | 22.6 ± 2.2 | 19.8 ± 4.6 |
| 20:3n-6 | 135 ± 28.2 | 138.4 ± 6.9 | 92.9 ± 17.4 |
| 20:4n-6 | 823.6 ± 140.4 | 770.8 ± 27.2 | 603.9 ± 26.2 |
| 22:4n-6 | 8 ± 1.9 | 7.2 ± 0.7 | 5.8 ± 0.8 |
| 22:5n-6 | 10.3 ± 1.2 | 9.1 ± 1.5 | 9.7 ± 0.3 |
| ***n-6 PUFAs*** | *3986.1 ± 0* | *3730.1 ± 0* | *3237.5 ± 0* |
| 18:3n-3 | 1 ± 0.2 | 1.5 ± 0.1 | 1.7 ± 0.9 |
| 20:5n-3 | 17.1 ± 4.3 | 11.3 ± 1.9 | 11.9 ± 2.1 |
| 22:5n-3 | 8.8 ± 1.8 | 6 ± 0.6 | 7 ± 0.4 |
| 22:6n-3 | 799.9 ± 144.8 | 642.3 ± 33.2 | 613.7 ± 35.1 |
| ***n-3 PUFAs*** | *826.8 ± 0* | *661.1 ± 0* | *634.2 ± 0* |

| **Supplemental Table 3H. Plasma fatty-acid concentrations of mice fed fish, algal and C^13^ enriched DHA diets at day 56** | | | |
| --- | --- | --- | --- |
|  | Concentration of fatty acids (nmol/ml) | | |
| Fatty acid | Fish-DHA (n=3) | Algal-DHA (n=4) | C^13^ Enriched-DHA (n=4) |
| 10:0 | 7.3 ± 3.1 | 4.2 ± 1.8 | 5.8 ± 2 |
| 12:0 | 330.1 ± 154 | 138.6 ± 55.6 | 231.5 ± 53.7 |
| 14:0 | 242.3 ± 102 | 156.1 ± 35.7 | 213.8 ± 36 |
| 16:0 | 2210.2 ± 824.2 | 2663.4 ± 282.7 | 2179.7 ± 122.3 |
| 18:0 | 1251.2 ± 499.5 | 1330.4 ± 133.4 | 1126.2 ± 65.9 |
| 20:0 | 35.9 ± 16.2 | 30.3 ± 4.6 | 39.3 ± 3.5 |
| 22:0 | 14.9 ± 5.1 | 17 ± 1.7 | 12.2 ± 1.2 |
| 24:0 | 6.2 ± 0.5 | 4.9 ± 0.6 | 3.4 ± 0.3 |
| ***SFAs*** | *4098 ± 0* | *4345 ± 0* | *3811.8 ± 0* |
| 14:1 | 5 ± 2 | 7.8 ± 1.2 | 3.5 ± 0.7 |
| 16:1n-7 | 167.2 ± 72.1 | 262.3 ± 58.5 | 170.6 ± 16.9 |
| 16:1n-9 | 18.9 ± 7.1 | 26.9 ± 4.3 | 21.1 ± 2.7 |
| 18:1n-7 | 83.3 ± 31.6 | 123.3 ± 17.8 | 110.2 ± 22.1 |
| 18:1n-9 | 806.6 ± 293.2 | 1161 ± 147.5 | 791.2 ± 81.1 |
| 20:1n-9 | 56.6 ± 29.7 | 50.3 ± 17.5 | 49.5 ± 7.9 |
| 22:1n-9 | 5.9 ± 2.4 | 5 ± 1.2 | 5 ± 0.6 |
| C 24:1n-9 | 2.5 ± 0.4 | 1.3 ± 0.2 | 1.3 ± 0.1 |
| ***MUFAs*** | *1146 ± 0* | *1637.9 ± 0* | *1152.2 ± 0* |
| 18:2n-6 | 3398 ± 1409.6 | 3460.3 ± 382.1 | 2946.5 ± 136.6 |
| 18:3n-6 | 34 ± 21.4 | 62.6 ± 9.2 | 37.4 ± 1.6 |
| 20:2n-6 | 13.7 ± 5.4 | 15.1 ± 0.8 | 20.9 ± 2 |
| 20:3n-6 | 80.4 ± 36.4 | 93.7 ± 11.7 | 93.5 ± 10.2 |
| 20:4n-6 | 736.4 ± 326.3 | 1137.7 ± 113.4 | 701.1 ± 34.3 |
| 22:4n-6 | 6.1 ± 2.7 | 10.3 ± 1.3 | 9.9 ± 1.3 |
| 22:5n-6 | 9.9 ± 3.4 | 14.9 ± 2.2 | 13.6 ± 1.7 |
| ***n-6 PUFAs*** | *4278.5 ± 0* | *4794.5 ± 0* | *3822.8 ± 0* |
| 18:3n-3 | 4.2 ± 0.7 | 1.5 ± 0.5 | 1.9 ± 0.2 |
| 20:5n-3 | 13.3 ± 5.6 | 10.9 ± 3.2 | 11.7 ± 1.4 |
| 22:5n-3 | 6 ± 2.3 | 7.1 ± 1.3 | 5.5 ± 0.8 |
| 22:6n-3 | 736.8 ± 281.7 | 804.3 ± 77.9 | 603.6 ± 21.7 |
| ***n-3 PUFAs*** | *760.4 ± 0* | *823.7 ± 0* | *622.8 ± 0* |

| **Supplemental Table 3I. Plasma fatty-acid concentrations of mice fed fish, algal and C^13^ enriched DHA diets at day 112** | | | |
| --- | --- | --- | --- |
|  | Concentration of fatty acids (nmol/ml) | | |
| Fatty acid | Fish-DHA (n=4) | Algal-DHA (n=4) | C^13^ Enriched-DHA (n=4) |
| 10:0 | 3 ± 0.5 | 3.6 ± 1.1 | 4.2 ± 1.3 |
| 12:0 | 274.4 ± 75.8 | 158.9 ± 43.3 | 235.3 ± 61.7 |
| 14:0 | 312 ± 51.8 | 198.3 ± 39.7 | 232 ± 55.3 |
| 16:0 | 3343 ± 257.5 | 2728.6 ± 216.1 | 2645.4 ± 446.9 |
| 18:0 | 1565.1 ± 56.3 | 1378.4 ± 43.8 | 1439.1 ± 206.1 |
| 20:0 | 51.3 ± 3.5 | 32.2 ± 2.2 | 49.5 ± 10.7 |
| 22:0 | 17.8 ± 0.6 | 11.4 ± 0.7 | 15.7 ± 2.9 |
| 24:0 | 5.5 ± 0.4 | 3.6 ± 0.2 | 4.8 ± 0.8 |
| ***SFAs*** | *5572.2 ± 0* | *4514.8 ± 0* | *4626 ± 0* |
| 14:1 | 12.4 ± 2.1 | 6.4 ± 0.6 | 7.2 ± 1.7 |
| 16:1n-7 | 271.9 ± 53.6 | 245 ± 31.5 | 151.9 ± 39.9 |
| 16:1n-9 | 36.1 ± 4.4 | 32.2 ± 3.3 | 26.9 ± 4.8 |
| 18:1n-7 | 147.5 ± 24.5 | 152.4 ± 13.3 | 106.5 ± 23.5 |
| 18:1n-9 | 1293.8 ± 141.5 | 1137.8 ± 115.1 | 881 ± 150.5 |
| 20:1n-9 | 61.1 ± 9 | 43.8 ± 4.3 | 40.7 ± 3.9 |
| 22:1n-9 | 8.9 ± 1.2 | 4.5 ± 0.3 | 5.5 ± 1 |
| 24:1n-9 | 1.4 ± 0.3 | 1.6 ± 0.5 | 1.7 ± 0.4 |
| ***MUFAs*** | *1833.2 ± 0* | *1623.7 ± 0* | *1221.4 ± 0* |
| 18:2n-6 | 4304 ± 209.9 | 3611.2 ± 286.2 | 3610.4 ± 428.2 |
| 18:3n-6 | 65.2 ± 1.9 | 51.7 ± 3.5 | 48.2 ± 5.3 |
| 20:2n-6 | 16.4 ± 1.6 | 15 ± 1.6 | 12 ± 1.5 |
| 20:3n-6 | 108.3 ± 20.6 | 129.6 ± 11.9 | 104 ± 15.1 |
| 20:4n-6 | 1014.5 ± 172.3 | 1191 ± 20.9 | 977.9 ± 114.7 |
| 22:4n-6 | 13.3 ± 1.1 | 11.7 ± 0.1 | 11.5 ± 2.9 |
| 22:5n-6 | 15 ± 0.9 | 11.9 ± 0.6 | 13.3 ± 3.3 |
| ***n-6 PUFAs*** | *5536.8 ± 0* | *5022.1 ± 0* | *4777.4 ± 0* |
| 18:3n-3 | 3.1 ± 0.5 | 2.5 ± 0.2 | 2.5 ± 0.2 |
| 20:5n-3 | 18.3 ± 4.8 | 18.9 ± 4.2 | 12.4 ± 1.5 |
| 22:5n-3 | 10.1 ± 1.2 | 8 ± 1.2 | 7.5 ± 1.1 |
| 22:6n-3 | 868.3 ± 100.8 | 720.9 ± 62.8 | 690.9 ± 78.6 |
| ***n-3 PUFAs*** | *899.8 ± 0* | *750.4 ± 0* | *713.3 ± 0* |

| **Supplemental Table 3J. Plasma fatty-acid concentrations of mice fed fish, algal and C^13^ enriched DHA diets at day 168** | | | |
| --- | --- | --- | --- |
|  | Concentration of fatty acids (nmol/ml) | | |
| Fatty acid | Fish-DHA (n=3) | Algal-DHA (n=4) | C^13^ Enriched-DHA (n=4) |
| 10:0 | 3.3 ± 2.6 | 0.7 ± 0.1 | 1.8 ± 1.2 |
| 12:0 | 150.1 ± 77.5 | 43.6 ± 15.8 | 109.7 ± 48.3 |
| 14:0 | 139.9 ± 56.5 | 49.9 ± 13.1 | 96.8 ± 31.7 |
| 16:0 | 1893.6 ± 447.5 | 1425.6 ± 99.8 | 1435.8 ± 248.6 |
| 18:0 | 882.3 ± 186.5 | 973.6 ± 76.6 | 807.5 ± 90.5 |
| 20:0 | 25.7 ± 6.2 | 20.8 ± 1.5 | 24.1 ± 2 |
| 22:0 | 10.1 ± 2.9 | 14.5 ± 3.9 | 13 ± 1.5 |
| 24:0 | 3.7 ± 1.7 | 5.6 ± 1 | 5.1 ± 0.4 |
| ***SFAs*** | *3108.7 ± 0* | *2534.3 ± 0* | *2493.7 ± 0* |
| 14:1 | 3.1 ± 1 | 0.8 ± 0.1 | 4.2 ± 1.8 |
| 16:1n-7 | 201.7 ± 62.3 | 85.6 ± 19.5 | 114.9 ± 25.3 |
| 16:1n-9 | 25.5 ± 8.4 | 14.9 ± 3.6 | 13.1 ± 2.8 |
| 18:1n-7 | 128.2 ± 54.1 | 109.3 ± 18 | 69.4 ± 8.8 |
| 18:1n-9 | 918 ± 307.3 | 527.9 ± 94.6 | 591.7 ± 115.5 |
| 20:1n-9 | 39 ± 10 | 23.4 ± 4.5 | 26.7 ± 4.7 |
| 22:1n-9 | 9.6 ± 4.8 | 5.5 ± 1.3 | 4.1 ± 0.7 |
| 24:1n-9 | 5 ± 1.3 | 8.4 ± 1.3 | 5.4 ± 0.6 |
| ***MUFAs*** | *1330.2 ± 0* | *775.8 ± 0* | *829.4 ± 0* |
| 18:2n-6 | 2628.7 ± 566.8 | 1626 ± 166.7 | 2044.9 ± 347.5 |
| 18:3n-6 | 14.7 ± 2.6 | 16.1 ± 1.4 | 16.3 ± 2.4 |
| 20:2n-6 | 7.6 ± 0.2 | 10.4 ± 1.5 | 7 ± 0.6 |
| 20:3n-6 | 110.8 ± 23.3 | 111.3 ± 7.8 | 72.1 ± 5.4 |
| 20:4n-6 | 754.1 ± 108.9 | 723.6 ± 107.9 | 587.2 ± 55.7 |
| 22:4n-6 | 8 ± 3.2 | 8.2 ± 2.2 | 7.3 ± 1 |
| 22:5n-6 | 10.1 ± 3 | 14.4 ± 4.5 | 10.3 ± 1.2 |
| ***n-6 PUFAs*** | *3534 ± 0* | *2510 ± 0* | *2745.1 ± 0* |
| 18:3n-3 | 2.8 ± 0.3 | 1.2 ± 0.1 | 2.2 ± 0.6 |
| 20:5n-3 | 22.5 ± 1.8 | 10.2 ± 1.7 | 10.5 ± 1.9 |
| 22:5n-3 | 10.7 ± 2.3 | 4.6 ± 0.5 | 6.9 ± 0.9 |
| 22:6n-3 | 701.8 ± 167.2 | 611.2 ± 29.8 | 610 ± 38.7 |
| ***n-3 PUFAs*** | *737.8 ± 0* | *627.2 ± 0* | *629.6 ± 0* |
